# Supplementary material for: Identification of Freezing-Responsive microRNAs and Their Targets in Chinese Jujube by Small RNA and Degradome Sequencing
Source: Int J Mol Sci. 2026 Sep 4;27(17):7912. doi: 10.3390/ijms27177912 (PMC13566890; doi:10.3390/ijms27177912)
Supplement: Supplementary file 1 [file ijms-27-07912-s001.zip › Supplementary Material S2/novel_miR_34.pdf]

Provisional ID	:	novel_miR_34
Score total	:	202059
Score for star read(s)	:	3.9
Score for read counts	:	202053
Score for mfe	:	0.5
Score for randfold	:	1.6
Score for cons. seed	:	
Total read count	:	396330
Mature read count	:	396251
Loop read count	:	0
Star read count	:	79

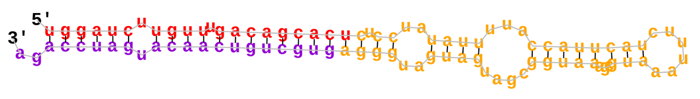

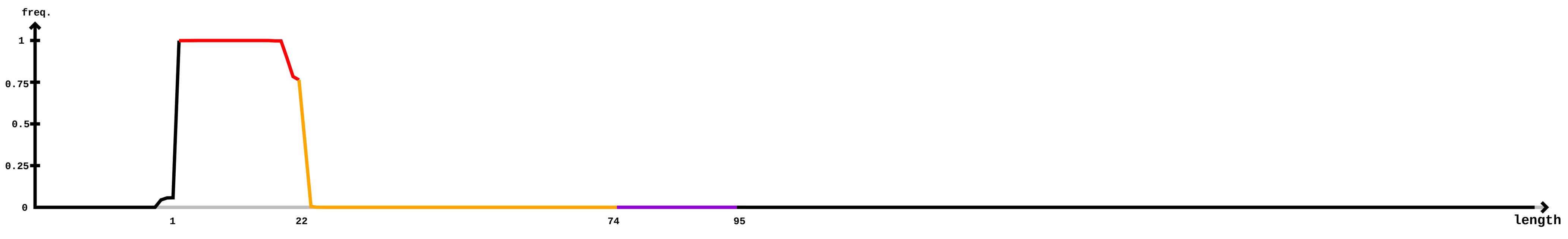

Mature Star

```
5'- uccaucuuuuucguuuacucucuggaucuuguuugacagcacucuccuauauuuuaccauucaucuuuaaaugcgaaauggcgaugaugaugggagugcugucaacaugauccagagggugcagucgaauaagauacagagggaccauccacaguaccuacucucaucacaaucuuauucuucuccucacuuauuauuuuucugaucauuuucuuuuacucuauugaugaaguguuauguuuugauucuu  -3'  obs
    uccaucuuuuucguuuacucucuggaucuuguuugacagcacucuccuauauuuuaccauucaucuuuaaaugcgaaauggcgaugaugaugggagugcugucaacaugauccagagggugcagucgaauaagauacagagggaccauccacaguaccuacucucaucacaaucuuauucuucuccucacuuauuauuuuucugaucauuuucuuuuacucuauugaugaaguguuauguuuugauucuu       exp
    ...(((((.((((((((((((((((((.((((.((((((((((.((..((((...((((((((......)))...)))))....))))..))))))))))))))))).))))))))))))).)).)))).))))).....((((.((...(((..((..(((.((((((...........................................................))))))))))..)))..)).)))).   reads  mm  sample
    ..caucuuuuucguuuacucuUu                 2    1   LT-3
    .............uuuacucucuggaucuug         1    0   JCK-3
    ...............uacucucuggaucuuguuugac   2    0   JCK-1
    ...............uacucucuggaucuuguuugac   1    0   JCK-2
    ...................cucuggaucuuguuugac   6    0   LCK-3
    ...................cucuggaucuuguuugac   13   0   LT-2
    ...................cucuggaucuuguuugac   4    0   JCK-1
    ...................cucuggaucuuguuugac   13   0   LT-3
    ...................cucuggaucuuguuugac   10   0   JCK-3
    ...................cucuggaucuuguuugac   13   0   JCK-2
    ...................cucuggCucuuguuugac   1    1   JCK-2
    ...................cucuggaucuuguuugac   8    0   LT-1
    ...................cucuggaucuuguuugaU   2    1   JCK-3
    ...................cucuggCucuuguuugaca  1    1   JT-1
    ...................cucuggaucuuguuugacC  1    1   LT-1
    ...................cucuggaucuuguuugaca  78   0   LCK-3
    ...................cucuggaucuuguuugaca  29   0   LCK-2
    ...................cucuggaucuuguuugacU  2    1   LT-1
    ...................cucuggaucuuguuugaca  20   0   JCK-1
    ...................cucuggaucuuguuugaca  18   0   JT-2
    ...................cucuggaucuuguuugaca  21   0   JT-3
    ...................cucuggaucuuguuugaca  62   0   LT-2
    ...................cucuggaucuuguuugaca  18   0   JCK-3
    ...................cucuggCucuuguuugaca  1    1   LCK-1
    ...................cuNuggaucuuguuugaca  1    1   LT-2
    ...................cucuggaucuuguuugacU  6    1   JCK-3
    ...................cucuggaucuuguuugaUa  1    1   JCK-3
    ...................cucuggaucuuguuugacU  6    1   LT-2
    ...................cucuggaucuuguuugaUa  1    1   JCK-2
    ...................cucuggaucuuguuugaca  42   0   LCK-1
    ...................cucuggaucuuguuugacU  14   1   LCK-3
    ...................cucuggaucuuguuugacU  7    1   JCK-2
    ...................cucuggaucuuguuugaca  28   0   JT-1
```

Mature Star

uccaucuuuuucguuuacucucuggaucuuguuugacagcacucuccuauauuuuaccauucaucuuuaaaugcgaaauggcgaugaugaugggagugcugucaacaugauccagagggugcagucgaauaagauacagagggaccauccacaguaccuacucucaucacaaucuuauucuucuccucacuuauuauuuuucugaucauuuucuuuuacucuauugaugaaguguuauguuuugauucuu

cucuggaucuuguuugaca	27	0	LT-1
cucuggaucuuguuugaca	65	0	LT-3
cucuggaucuuguuugacU	1	1	LT-3
cucuggaucuuguuugaca	41	0	JCK-2
cucuggaucuuguuugacag	5	0	JCK-1
cucuggaucuuguuugacaU	2	1	LT-1
cucuggaucuuguuugacag	23	0	LCK-3
cucuggaucuuAuuugacag	1	1	LT-2
cucuggaucuuguuugacag	12	0	LT-2
cucuggaucuuguuugacaU	3	1	LT-3
cucuggaucuuguuugacag	7	0	LT-1
cucuggaucuuguuugacag	15	0	JT-3
cucuggaucuuguuugacag	14	0	JT-2
cucuggaucuuguuugacag	7	0	LT-3
cucuggaucuuguuugacag	4	0	JCK-2
cucuggaucuuguuugacag	6	0	JCK-3
cucuggauNuuguuugacagc	2	1	JCK-3
cuUggaucuuguuugacagc	3	1	JCK-3
cucCggaucuuguuugacagc	2	1	JCK-3
cucuggaucuuguuugacagc	1675	0	LT-2
cucCggaucuuguuugacagc	2	1	JCK-2
cucGggaucuuguuugacagc	1	1	JCK-1
cucuggaucuuguuugacCgc	2	1	JCK-3
cucuUgaucuuguuugacagc	1	1	LT-2
cucuggaucuuAuuugacagc	2	1	JCK-2
cucCggaucuuguuugacagc	1	1	LT-2
cucuggCucuuguuugacagc	4	1	LCK-3
cNcuggaucuuguuugacagc	2	1	JCK-1
cucuggCucuuguuugacagc	11	1	LT-3
cucuggaucuuguuugacagc	292	0	LCK-2
cucuggaucuuguuugacagc	275	0	LCK-1
cucuggaucuuguCugacagc	1	1	JCK-3
cucugAaucuuguuugacagc	3	1	LT-2
cucuggaucCuguuugacagc	1	1	LT-1
cNcuggaucuuguuugacagc	4	1	JCK-2
cucuggaucuuguuugacagA	3	1	LT-2
cucuggaucuCguuugacagc	1	1	LT-1
cNcuggaucuuguuugacagc	1	1	LCK-1
cucuggaucuuUuuugacagc	1	1	LT-1
cucuggaucuuguuugacCgc	2	1	LCK-3
cuUggaucuuguuugacagc	2	1	JCK-1
cucuggaucuuguuugCcagc	1	1	JCK-3
cucuggCucuuguuugacagc	1	1	JCK-1
cuAuggaucuuguuugacagc	2	1	JCK-3
cucuggaucuuguuugaUagc	1	1	LT-3
cucugNaucuuguuugacagc	1	1	LCK-1
cucuggaCcuuguuugacagc	2	1	LT-1
cucuggaucuuguuugCcagc	5	1	LT-3
cCcuggaucuuguuugacagc	1	1	LT-1
cucCggaucuuguuugacagc	1	1	LT-1
cucuggCucuuguuugacagc	11	1	LT-1
cCcuggaucuuguuugacagc	1	1	JCK-2
cucuggaucuuguuugacagc	66	0	JT-1
cucuggaucuuguuuGcagc	1	1	JCK-3
cucuggaucuuguuugacGgc	1	1	LT-3
cucuggaucuCguuugacagc	1	1	LCK-3
cucuggCucuuguuugacagc	11	1	JCK-2
cNcuggaucuuguuugacagc	1	1	JCK-3
cucuggaucuuNuuugacagc	3	1	JCK-1
cucuggaucuuguuugCcagc	9	1	LT-1
cucuggaucuuguuugacagc	1680	0	JCK-1
cucuggaucuuguuugCcagc	2	1	LCK-3
cucuggCucuuguuugacagc	2	1	LCK-1
cuNuggaucuuguuugacagc	2	1	LT-2
cucuUgaucuuguuugacagc	1	1	LT-3
cucugAaucuuguuugacagc	1	1	LT-3
cucuggaucuuguuugacagU	2	1	LT-3
cucuAgaucuuguuugacagc	1	1	JCK-1
cucuggaucCuguuugacagc	1	1	JCK-2
cucuggaucuuguuuUacagc	1	1	JCK-2

Mature **Star**

uccaucuuuuucguuuacucucuggaucuuguuugacagcacucuccuauauuuuaccauucaucuuuaaaugcgaaauggcgaugaugaugggagugcugucaacaugauccagagggugcagucgaauaagauacagagggaccauccacaguaccuacucucaucacaaucuuauucuucuccucacuuauuauuuuucugaucauuuucuuuuacucuauugaugaaguguuauguuuugauucuu

...................cCcuggaucuuguuugacagc	2	1	LT-2
...................cucuggaucuuguuugacagc	2088	0	JCK-3
...................cucuggaucuuguuugCcagc	3	1	JCK-2
...................cucuggaucuuAuuugacagc	1	1	LT-1
...................cucuAgaucuuguuugacagc	3	1	LT-3
...................cucuggaucuuguuugacCgc	4	1	LT-3
...................cucuggaucuuguuuAacagc	1	1	LCK-3
...................cucuggaucuuguuugacagc	3001	0	LT-3
...................cucuggaucuCguuugacagc	2	1	LT-2
...................cucuggaucuuguuugaUagc	2	1	JCK-2
...................cucuggaucuuguuugacagc	3319	0	LT-1
...................cucuggCucuuguuugacagc	4	1	JCK-3
...................cucuggCucuuguuugacagc	20	1	LT-2
...................cucuggaucuuguuugacagc	1379	0	LCK-3
...................cucuggaucuuguuugacCgc	2	1	JCK-2
...................cucugUaucuuguuugacagc	1	1	LT-1
...................cucuggaucuuguuugCcagc	4	1	JCK-1
...................cNcuggaucuuguuugacagc	1	1	LT-3
...................cucuggaucuuguuugacagc	2907	0	JCK-2
...................cucuggaucuuguCugacagc	1	1	JCK-2
...................cucuggaucuuguuugacCgc	2	1	LT-2
...................cucuAgaucuuguuugacagc	1	1	LCK-3
...................cucuggaucuuguuugacagc	90	0	JT-3
...................cucugAaucuuguuugacagc	1	1	JCK-3
...................cucuggaucuuguuugacagc	62	0	JT-2
...................cucuggCucuuguuugacagc	1	1	LCK-2
...................cucuggaucuuguuugacaNc	4	1	JCK-1
...................cucuggaucuuguuugaUagc	2	1	JCK-3
...................cuGuggaucuuguuugacagc	1	1	LT-2
...................cucNggaucuuguuugacagc	2	1	JCK-1
...................cucCggaucuuguuugacagc	3	1	LT-3
...................cucuAgaucuuguuugacagc	4	1	LT-2
...................cucuggaucuuUuuugacagc	1	1	JCK-3
...................cucuggaucuuguuugacagU	1	1	JCK-3
...................cucuggaucuuAuuugacagc	1	1	LT-3
...................cGcuggaucuuguuugacagc	1	1	LT-2
...................cucuggaucuuguuugCcagc	7	1	LT-2
...................cCcuggaucuuguuugacagc	1	1	LCK-3
...................cucuAgaucuuguuugacagc	1	1	JCK-2
...................cucuggauAuuguuugacagc	1	1	LT-1
...................cucuggaucuuguuuAacagc	1	1	LT-1
...................cucuggaucuugCuugacagc	1	1	JCK-2
...................cucuggaucuuguuugacCgc	2	1	JCK-1
...................cucuggauUuuguuugacagc	1	1	LT-1
...................cucuggaucuuguuugacCgc	2	1	LT-1
...................cCcuggaucuuguuugacagc	1	1	LCK-2
...................cucuggaucuuguuugaGagc	1	1	JCK-1
...................cucuggaucuuguuugacagcC	1	1	JCK-2
...................cucuggaucuuguuugacagca	22	0	LT-3
...................cucuggaucuuguuugacagca	1	0	JT-2
...................cucuggaucuuguuugacagca	87	0	JCK-3
...................cucuggaucuuguuugacagcU	14	1	JT-3
...................cucuggaucuuguuugacagcU	69	1	LT-3
...................cucuggaucuuguuugacagcU	55	1	JCK-2
...................cucuggaucuuguuugacagcC	2	1	LT-1
...................cucuggaucuuguuugacagcU	29	1	JCK-3
...................cucuggaucuuguuugacagcU	66	1	LT-1
...................cucuggaucuuguuugacagcC	2	1	LT-3
...................cucuggaucuuguuugacagca	10	0	JT-3
...................cucuggaucuuguuugacagca	19	0	LT-2
...................cucuggaucuugCuugacagca	1	1	JCK-2
...................cucuggaucuuguuugacagcC	2	1	LT-2
...................cucuggaucuuguuugacagcU	84	1	LCK-3
...................cucuggaucuuguuuNacagca	1	1	JCK-3
...................cucuggaucuuguuugacagcU	47	1	LT-2
...................cucuggaucuuguuugacagcU	13	1	JT-2
...................cucuggaucuuguuugacagcU	25	1	JCK-1
...................cucuggaucuuguuugacagca	5	0	LCK-3
...................cucuggaucuuguuugacagca	72	0	JCK-1
...................cucuggaucuuguuugacagca	27	0	LT-1

Mature Star

uccaucuuuuucguuuacucucuggaucuuguuugacagcacucuccuauauuuuaccauucaucuuuaaaugcgaaauggcgaugaugauggga**gugcugucaacaugauccaga**gggugcagucgaauaagauacagagggaccauccacaguaccuacucucaucacaaucuuauucuucuccucacuuauuauuuuucugaucauuuucuuuuacucuauugaugaaguguuauguuuugauucuu			
...................cucuggaucuuguuugacagcG	1	1	JT-2
...................cucuggaucuuguuugacagca	117	0	JCK-2
...................cucuggaucuuguuugacagcac	1	0	LT-3
...................cucuggaucuuguuugacagcac	1	0	LT-1
...................cucuggaucuuguuugacagcacu	1	0	LT-2
...................cucuggaucuuguuugacagcacN	1	1	LT-2
....................ucuggaucuuguuugaca	33	0	JCK-2
....................ucuggaucuuguuugaca	9	0	JT-1
....................ucuggaucuuguuugaca	25	0	JCK-1
....................ucuggaucuuguuugaca	28	0	LT-1
....................ucuggaucuuguuugaca	26	0	LT-3
....................ucuggaucuuguuugaca	79	0	LCK-3
....................ucuggaucuuguuugaca	15	0	JCK-3
....................ucuggaucuuguuugaca	3	0	LCK-2
....................ucuggaucuuguuugaca	3	0	LCK-1
....................ucuggaucuuguuugaca	30	0	JT-2
....................ucuggaucuuguuugacU	9	1	LCK-3
....................ucuggaucuuguuugacU	2	1	LT-3
....................ucuggaucuuguuugacU	6	1	LT-1
....................ucuggaucuuguuugaca	41	0	LT-2
....................ucuggaucuuguuugacU	1	1	JCK-2
....................ucuggaucuuguuugaca	35	0	JT-3
....................ucuggaucuuguuugacU	1	1	JCK-1
....................ucuggaucuuguuugacag	2	0	LT-1
....................ucuggaucuuguuugacagc	127	0	LT-3
....................ucuggaucuuguuugacagA	1	1	LT-3
....................ucuggaucuuguuuNacagc	1	1	LCK-1
....................ucuggaucuuguuugacagc	29	0	JCK-1
....................uGuggaucuuguuugacagc	1	1	LCK-1
....................ucuggaucuuguuugacagc	55	0	JCK-2
....................ucuggaucuuguuugacagc	41	0	LCK-3
....................ucuggaucuuguuugacagA	1	1	JCK-3
....................ucuggCucuuguuugacagc	1	1	LCK-1
....................ucuggaucuuguuugacagc	35	0	JCK-3
....................ucuggaucuuguuuUacagc	1	1	LCK-2
....................ucuggaucuuguuugacagc	75	0	LCK-2
....................ucuggaucuuguuugacagA	1	1	JCK-1
....................ucuggaucuuguuugacagc	63	0	LCK-1
....................ucuggCucuuguuugacagc	2	1	LT-2
....................ucuggaucuuguuugacagc	116	0	LT-2
....................ucuggaucuuguuugacaUc	1	1	LT-1
....................ucuggaucuuguuugacagc	107	0	LT-1
....................ucuggaucuuguuugacagca	267	0	LT-3
....................ucuggaucuuguuugacagcU	14	1	LT-2
....................uNuggaucuuguuugacagca	1	1	JCK-1
....................ucuggaucuuguuugacagcU	3	1	JCK-3
....................ucuggaucuuguuugacagca	17	0	LCK-2
....................uUuggaucuuguuugacagca	1	1	JCK-1
....................ucuggaucuuguuugacagca	12	0	JT-3
....................uUuggaucuuguuugacagca	8	1	JCK-3
....................ucuggauUuuguuugacagca	1	1	JCK-2
....................ucuggaucuuguuugCcagca	2	1	LT-3
....................ucuggaucuuguuugacagcU	6	1	JCK-1
....................ucuggauAuuguuugacagca	1	1	JCK-2
....................ucuggaucuCguuugacagca	1	1	LT-3
....................ucuggaucuuguuugacagca	9	0	LCK-1
....................ucuggaucuuguuugacaUca	3	1	JCK-3
....................ucuggaucuuguuugacagca	832	0	JCK-3
....................ucuggaucuuguuugUcagca	1	1	JCK-3
....................ucCggaucuuguuugacagca	1	1	JCK-1
....................uUuggaucuuguuugacagca	12	1	JCK-2
....................ucuNgaucuuguuugacagca	1	1	JCK-1
....................ucuggaucuuguuugacaUca	5	1	JCK-2
....................ucuggaucuuguuugacagcU	6	1	LT-1
....................ucuggaucuuguuugCcagca	1	1	LT-1
....................ucCggaucuuguuugacagca	1	1	JCK-2
....................ucuggaucuuguuugacagca	29	0	JT-1
....................ucuggaucCuguuugacagca	1	1	JCK-2
....................ucuggaucuuguuugCcagca	1	1	JCK-1
....................ucuggaucuuguuugacagca	145	0	LCK-3

Mature Star

```
uccaucuuuuucguuuacucucuggaucuuguuugacagcacucuccuauauuuuaccauucaucuuuaaaugcgaaauggcgaugaugauggga gugcugucaacaugauccagagggugcagucgaauaagauacagagggaccauccacaguaccuacucucaucacaaucuuauucuucuccucacuuauuauuuuucugaucauuuucuuuuacucuauugaugaaguguuauguuuugauucuu
....................ucuggaucuuguuugacagcN.........   1     1   JCK-1
....................ucuggaucuuguuugacagcU.........   3     1   JCK-2
....................ucuggaucuuguuugacagca.........   1097  0   JCK-2
....................ucuggaucuuguuugacagcU.........   7     1   LT-3
....................ucuggaucuuguuugacagcU.........   9     1   LCK-3
....................ucuggaucuuguuugacGgca.........   2     1   JCK-1
....................ucuggaucuugGuugacagca.........   1     1   JCK-1
....................ucuggaucuuguuugGcagca.........   1     1   LT-2
....................ucuggaucuuguuugacagca.........   628   0   JCK-1
....................ucuggCucuuguuugacagca.........   1     1   JCK-2
....................ucuggaucuuguuugacagNa.........   2     1   JCK-1
....................ucuggaucuuguuugacagcC.........   1     1   LCK-3
....................ucuggaucuuguuugGcagca.........   1     1   LCK-3
....................ucuggaucuuguuugacagca.........   195   0   LT-2
....................ucuggaucuugGuugacagca.........   2     1   LT-1
....................ucuggaucuuguuugacagca.........   11    0   JT-2
....................ucuggaucuuguuugUcagca.........   1     1   JCK-2
....................ucuggaucuuguuugacaUca.........   2     1   JCK-1
....................ucuggaucuuguuugacagUa.........   1     1   JCK-2
....................ucuggaucuuguuugacagca.........   299   0   LT-1
....................ucuggaucuuguuugacagcac........   34    0   JCK-1
....................ucuggaucuuguuugacagcac........   24    0   LT-3
....................ucuggaucuuguuugacagcac........   27    0   LT-1
....................ucuggaucuuguuugacagcac........   19    0   LCK-3
....................ucuggaucuuguuugacagcac........   50    0   JCK-2
....................ucuggaucuuguuugacagcac........   17    0   LT-2
....................ucuggaucuuguuugacagcaU........   1     1   LT-1
....................ucuggaucuuguuugacagcaU........   2     1   LT-2
....................ucuggaucuuguuugacagcac........   22    0   JCK-3
....................ucuggaucuuguuugCcagcac........   1     1   LT-2
....................ucuggaucuuguuugacagcacu.......   2     0   JCK-3
....................ucuggaucuuguuugacagcacu.......   2     0   JCK-1
....................ucuggaucuuguuugacagcacu.......   3     0   LT-1
....................ucuggaucuuguuugacagcacu.......   5     0   JCK-2
....................ucuggaucuuguuugacagcaUu.......   1     1   LT-3
....................ucuggaucuuguuugacagcacuc......   1     0   JCK-1
....................ucuggaucuuguuugacagcacuc......   2     0   LT-3
....................ucuggaucuuguuugacagcacuc......   1     0   JCK-3
....................ucuggaucuuguuugacagcacuc......   1     0   JCK-2
....................ucuggaucuuguuugacagcacucu.....   2     0   LT-1
.....................cuggaucuuguuugacagc..........   5     0   LCK-3
.....................cuggaucuuguuugacagc..........   2     0   JCK-3
.....................cuggaucuuguuugacagc..........   1     0   JCK-1
.....................cuggaucuuguuugacagc..........   7     0   JCK-2
.....................cuggaucuuguuugacagc..........   1     0   LT-3
.....................cuggaucuuguuugacagc..........   5     0   LT-1
.....................cuggaucuuguuugacagc..........   13    0   LT-2
.....................cuggaucuuguuugacagca.........   11    0   LT-2
.....................cuggaucuuguuugacagca.........   58    0   JCK-1
.....................cuggaucuuguuugacagca.........   9     0   LT-1
.....................cuggaucuuguuugacagcU.........   5     1   LT-2
.....................cuggaucuuguuugacagcU.........   4     1   JT-2
.....................cuggaucuuguuugacagcU.........   4     1   JT-3
.....................cuggaucuuguuugacagcU.........   1     1   JCK-2
.....................cuggaucuuguuugacagca.........   61    0   JCK-2
.....................cuggaucuuguuugacagca.........   15    0   LT-3
.....................cuggaucuuguuugacagca.........   51    0   JCK-3
.....................cuggaucuuguuugacagcU.........   1     1   LT-3
.....................cuggaucuuguuugCcagca.........   1     1   JCK-1
.....................cuggaucuuguuugacagcU.........   1     1   JCK-3
.....................cuggaucuuguuugacagca.........   12    0   LCK-3
.....................cuggaucuuguuugacagcac........   4     0   LT-2
.....................cuggaucuuguuugacagcac........   3     0   LT-3
.....................cuggaucuuguuugacagcac........   1     0   LCK-3
.....................cuggaucuuguuugacagcacu.......   13    0   LT-3
.....................cuggaucuuguuugacagcacu.......   17    0   JCK-1
.....................cuggaucuuguuugacagcacu.......   22    0   JCK-3
.....................cuggaucuuguuugacagcacu.......   15    0   LCK-3
.....................cuggaucuuguuugacagcaUu.......   2     1   JCK-3
.....................cuggaucuuguuugacagcacu.......   4     0   LT-1
```

```
                      Mature                                                                 Star
uccaucuuuuucguuuacucucuggaucuuguuugacagcacucuccuauauuuuaccauucaucuuuaaaugcgaaauggcgaugaugaugggagugcugucaacaugauccagagggugcagucgaauaagauacagagggaccauccacaguaccuacucucaucacaaucuuauucuucuccucacuuauuauuuuucugaucauuuucuuuuacucuauugaugaaguguuauguuuugauucuu
.....................cuggaucuuguuugacagcacu..............................................................................................................................................................................................   7      0   LT-2
.....................cuggaucuuguuugacagcacu..............................................................................................................................................................................................   46     0   JCK-2
.....................cuggaucuuguuugacagcaUu..............................................................................................................................................................................................   1      1   JCK-2
.....................cuggaucuuguuugacagcacuc.............................................................................................................................................................................................   3      0   JCK-3
.....................cuggaucuuguuugacagcacuc.............................................................................................................................................................................................   1      0   JCK-1
.....................cuggaucuuguuugacagcacuc.............................................................................................................................................................................................   1      0   JCK-2
.....................cuggaucuuguuugacagcacucu............................................................................................................................................................................................   3      0   JCK-2
.....................cuggaucuuguuugacagcacucuc...........................................................................................................................................................................................   1      0   JCK-1
.....................cuggaucuuguuugacagcacucuc...........................................................................................................................................................................................   3      0   JCK-2
......................uggaucuuguuugacagA.................................................................................................................................................................................................   6      1   LT-3
......................uggaucuuguuugacagA.................................................................................................................................................................................................   7      1   LT-2
......................uggaucuuguuugacagc.................................................................................................................................................................................................   2328   0   LT-3
......................uggaucuuguuugCcagc.................................................................................................................................................................................................   2      1   LCK-2
......................uNgaucuuguuugacagc.................................................................................................................................................................................................   1      1   LT-3
......................uggaucuuguuugacagA.................................................................................................................................................................................................   1      1   LCK-1
......................uNgaucuuguuugacagc.................................................................................................................................................................................................   3      1   JCK-2
......................uggaucCuguuugacagc.................................................................................................................................................................................................   1      1   LT-3
......................uggCucuuguuugacagc.................................................................................................................................................................................................   5      1   LT-1
......................uggaucuuguuugacagc.................................................................................................................................................................................................   520    0   JT-1
......................uAgaucuuguuugacagc.................................................................................................................................................................................................   4      1   LCK-3
......................ugAaucuuguuugacagc.................................................................................................................................................................................................   1      1   LT-3
......................uggCucuuguuugacagc.................................................................................................................................................................................................   3      1   LCK-2
......................uggaucuuguuugacagc.................................................................................................................................................................................................   1447   0   LCK-1
......................uggaucuuguuugacagU.................................................................................................................................................................................................   1      1   JCK-3
......................uggaucuuAuuugacagc.................................................................................................................................................................................................   3      1   LT-2
......................uggGucuuguuugacagc.................................................................................................................................................................................................   1      1   LCK-3
......................uggaucuuguuugacagc.................................................................................................................................................................................................   5296   0   LT-2
......................uggaucuuguuugacagU.................................................................................................................................................................................................   3      1   JCK-2
......................uggaucuuguuugacagA.................................................................................................................................................................................................   3      1   JCK-1
......................uggaucuuguuugacagA.................................................................................................................................................................................................   1      1   JCK-3
......................uggaucuuguuugacCgc.................................................................................................................................................................................................   2      1   LT-1
......................uggaucuuguuNgacagc.................................................................................................................................................................................................   1      1   JCK-1
......................uggaucuuguuugacagU.................................................................................................................................................................................................   13     1   LCK-3
......................uggaucuuguuugaNagc.................................................................................................................................................................................................   11     1   LCK-1
......................uggCucuuguuugacagc.................................................................................................................................................................................................   7      1   LT-3
......................uggaucuuguuugacagG.................................................................................................................................................................................................   1      1   JCK-1
......................uggCucuuguuugacagc.................................................................................................................................................................................................   3      1   JT-1
......................uggaucuuguuugacagU.................................................................................................................................................................................................   12     1   LT-3
......................uggaucuuguuugNcagc.................................................................................................................................................................................................   6      1   LCK-1
......................uggaucuuguuugacagc.................................................................................................................................................................................................   1573   0   LCK-2
......................ugUaucuuguuugacagc.................................................................................................................................................................................................   1      1   JCK-1
......................uggCucuuguuugacagc.................................................................................................................................................................................................   2      1   LCK-1
......................uNgaucuuguuugacagc.................................................................................................................................................................................................   1      1   JCK-1
......................uggCucuuguuugacagc.................................................................................................................................................................................................   1      1   JCK-3
......................uggaucuuguuugacaCc.................................................................................................................................................................................................   2      1   JCK-3
......................uggaucuuguuNgacagc.................................................................................................................................................................................................   1      1   JCK-2
......................ugAaucuuguuugacagc.................................................................................................................................................................................................   1      1   JCK-2
......................uggaucuugCuugacagc.................................................................................................................................................................................................   1      1   LT-1
......................uggCucuuguuugacagc.................................................................................................................................................................................................   1      1   JCK-1
......................ugAaucuuguuugacagc.................................................................................................................................................................................................   3      1   LT-2
......................uggaucuuguuugacagc.................................................................................................................................................................................................   1255   0   JCK-3
......................uggCucuuguuugacagc.................................................................................................................................................................................................   53     1   LT-2
......................uggaucuuguuugCcagc.................................................................................................................................................................................................   6      1   LT-1
......................ugAaucuuguuugacagc.................................................................................................................................................................................................   1      1   LT-1
......................uggaucuuguuugacagc.................................................................................................................................................................................................   353    0   JT-2
......................ugAaucuuguuugacagc.................................................................................................................................................................................................   2      1   LCK-1
......................uggauNuuguuugacagc.................................................................................................................................................................................................   1      1   LCK-1
......................uCgaucuuguuugacagc.................................................................................................................................................................................................   1      1   LT-1
......................ugUaucuuguuugacagc.................................................................................................................................................................................................   1      1   LCK-3
......................uggaucuuguuugCcagc.................................................................................................................................................................................................   15     1   LCK-3
......................uggaucuuguuugacCgc.................................................................................................................................................................................................   2      1   LCK-2
......................uggaucuuguuugacCgc.................................................................................................................................................................................................   1      1   JT-1
......................uggaucuuguuugacCgc.................................................................................................................................................................................................   2      1   LCK-3
......................uggaucuuAuuugacagc.................................................................................................................................................................................................   1      1   LCK-2
......................uNgaucuuguuugacagc.................................................................................................................................................................................................   1      1   JT-1
......................Cggaucuuguuugacagc.................................................................................................................................................................................................   1      1   LT-3
......................uggaucuuguuugacagc.................................................................................................................................................................................................   6351   0   LCK-3
......................uggGucuuguuugacagc.................................................................................................................................................................................................   1      1   LT-2
......................uggaucuAguuugacagc.................................................................................................................................................................................................   1      1   LT-1
......................uggaucuuguuuAacagc.................................................................................................................................................................................................   1      1   LCK-3
```

Mature **Star**

uccaucuuuuucguuuacucucuggaucuuguuugacagcacucuccuauauuuuaccauucaucuuuaaaugcgaaauggcgaugaugaugggagugcugucaacaugauccagagggugcagucgaauaagauacagagggaccauccacaguaccuacucucaucacaaucuuauucuucuccucacuuauuauuuuucugaucauuuucuuuuacucuauugaugaaguguuauguuuugauucuu

......................uggNucuuguuugacagc	1	1	JCK-1
......................uAgaucuuguuugacagc	4	1	LT-3
......................uggaucuuguuugacagU	1	1	JCK-1
......................uggCucuuguuugacagc	2	1	JT-3
......................uNgaucuuguuugacagc	1	1	LCK-3
......................uggaucuuguuugCcagc	24	1	LT-2
......................uggaucuCguuugacagc	1	1	LT-1
......................uggaucuuguuugacagU	21	1	LT-2
......................uUgaucuuguuugacagc	1	1	LT-2
......................uggaucuuguuugacagc	419	0	JT-3
......................uggaucuugCuugacagc	1	1	JCK-1
......................uggaucuuAuuugacagc	1	1	LT-3
......................ugUaucuuguuugacagc	1	1	LT-2
......................uAgaucuuguuugacagc	1	1	LT-1
......................uggaucuuguuugacCgc	6	1	LT-2
......................uNgaucuuguuugacagc	2	1	LCK-1
......................uggaucuugCuugacagc	1	1	JCK-2
......................uggaucuuguuugacagc	1810	0	JCK-2
......................uggaucuuguuuUacagc	1	1	JT-2
......................uggaucuuguuugacagc	1080	0	JCK-1
......................uggaucuuguuugGcagc	2	1	LCK-1
......................uNgaucuuguuugacagc	1	1	JCK-3
......................ugAaucuuguuugacagc	3	1	LCK-3
......................uggaucuuAuuugacagc	1	1	JCK-1
......................uUgaucuuguuugacagc	1	1	JCK-2
......................uggaucuuguuugCcagc	1	1	JCK-3
......................uggCucuuguuugacagc	2	1	JCK-2
......................uAgaucuuguuugacagc	1	1	LCK-2
......................uggaucuuguCugacagc	2	1	JCK-2
......................uggaucuuguuugacCgc	2	1	JCK-2
......................uUgaucuuguuugacagc	1	1	LT-3
......................uggNucuuguuugacagc	1	1	JT-2
......................uggaucuuguuugCcagc	4	1	LT-3
......................uUgaucuuguuugacagc	1	1	LCK-3
......................uggaucuuguuuAacagc	1	1	LCK-2
......................Cggaucuuguuugacagc	1	1	LT-2
......................ugNaucuuguuugacagc	1	1	LCK-1
......................uggaucuuguuuNacagc	3	1	LCK-1
......................uggCucuuguuugacagc	10	1	LCK-3
......................uggaucuuguuugCcagc	2	1	LCK-1
......................ugNaucuuguuugacagc	7	1	LT-2
......................uggaucuuguuugacGgc	1	1	LT-2
......................uggaucuuguuugacagU	9	1	LT-1
......................uggaucuuguuNgacagc	1	1	JT-1
......................uggaucuuguuugCcagc	3	1	JCK-1
......................uggaucuuAuuugacagc	3	1	LT-1
......................uggaucuuguuugCcagc	4	1	JCK-2
......................uggaucuuguuugacagA	4	1	LCK-3
......................uggaucuuguGugacagc	1	1	LT-2
......................uggCucuuguuugacagc	2	1	JT-2
......................uggaucuuguuugacagc	2286	0	LT-1
......................uggaucuuguuugaGagc	4	1	LCK-1
......................uAgaucuuguuugacagc	5	1	LT-2
......................uggaucuuguuugacagca	1154	0	JCK-1
......................uggaucuuguuNgacagca	5	1	JCK-1
......................uggaucuuguuugacagca	1426	0	JT-1
......................uggaucuuUuuugacagca	1	1	LCK-2
......................uggaucuCguuugacagca	1	1	LT-1
......................uggCucuuguuugacagca	2	1	JT-3
......................Cggaucuuguuugacagca	1	1	LCK-3
......................uggaucuuguuugacagUa	1	1	LCK-2
......................uggaCcuuguuugacagca	1	1	LT-2
......................uggauNuuguuugacagca	10	1	LCK-1
......................ugNaucuuguuugacagca	5	1	LCK-1
......................uggaucuuguuugCcagca	3	1	LCK-1
......................uggaucuuguuugacagcU	718	1	JCK-1
......................uggaucuuguuugacagcU	2081	1	LT-3
......................uggCucuuguuugacagca	5	1	LCK-1
......................uggaucuCguuugacagca	1	1	JT-1
......................uggaucuuguuuUacagca	1	1	LCK-3

Mature **Star**

uccaucuuuuucguuuacucucuggaucuuguuugacagcacucuccuauauuuuaccauucaucuuuaaaugcgaaauggcgaugaugaugggga gugcugucaacaugauccagagggugcagucgaauaagauacagagggaccauccacaguaccuacucucaucacaaucuuauucuucuccucacuuauuauuuuucugaucauuuucuuuuacucuauugaugaaguguuauguuuugauucuu

uggaucuuguuugacagcG	2	1	JCK-2
uggaucuuguuNgacagca	2	1	JT-1
uggaucuuguuugacagcU	1372	1	LCK-1
uggaucuuguuugacCgca	1	1	JCK-1
uggaucuuguuugaNagca	11	1	LCK-1
uggaucuugCuugacagca	1	1	JT-1
uggaucuuguuugacagca	1862	0	JCK-2
uggaucuuguuNgacagca	1	1	JT-2
uggaucuuguuugacagca	1490	0	LT-3
uggaucuuguuugaAagca	1	1	LCK-3
uAgaucuuguuugacagca	1	1	LCK-2
uggaucuuguuGgacagca	9	1	LCK-3
uggaucuuguuugacagca	2333	0	LT-2
uggaucuuguuugacagUa	10	1	JT-3
uggaucuuguuugacagcC	21	1	LCK-3
uggaucuuguuugCcagca	1	1	LT-1
uggaucuuguuugaAagca	1	1	LCK-2
uggaucuuguuugacagcC	14	1	LT-1
uggaucuuAuuugacagca	1	1	LCK-3
uggaucuuguuugacCgca	2	1	LCK-1
uggaucuuguuugNcagca	9	1	LCK-1
uggGucuuguuugacagca	1	1	LCK-2
uggaucuuguuugGcagca	2	1	LCK-1
uggaucuuguuugacagUa	29	1	LT-2
uggaucCuguuugacagca	1	1	LT-3
uggaucuuguuugacagcU	381	1	JT-3
uggCucuuguuugacagca	3	1	JCK-3
uggaucuuguuugacagcC	2	1	JCK-1
uggaucuuguuugacagcG	15	1	LT-2
uggaucuuguuugCcagca	2	1	LCK-3
uggaucuuguuugaGagca	4	1	LCK-1
uggaucuuguuuNacagca	2	1	LCK-1
uggaucuuguuugacagca	3565	0	LCK-1
uggaucuuguuugacagUa	3	1	LT-1
uggaucuuguuugacCgca	1	1	LT-2
uggaucuuguuugacagcU	4465	1	LCK-3
uggaucuuguuugGcagca	1	1	LT-2
uggaucuuguuugacagcU	735	1	JT-1
uNgaucuuguuugacagca	2	1	JCK-1
uggaucuuguuugacagcC	6	1	JCK-3
uggaucuuguuugacagcU	2784	1	LT-2
uggaucuuguuugacagUa	18	1	LCK-3
uggaucuuguuugacagcG	2	1	JT-2
uNgaucuuguuugacagca	1	1	JCK-3
uggCucuuguuugacagca	4	1	JCK-2
uggaucuuguuugacagcC	20	1	LT-2
ugAaucuuguuugacagca	1	1	LT-3
uggaucuuguuugacagcG	12	1	LT-1
uggaucuuguuugacagcU	835	1	JCK-3
uNgaucuuguuugacagca	2	1	JCK-2
uggaucuuguuugacagca	858	0	LT-1
uggauUuuguuugacagca	1	1	LT-2
uggaucuuguuugacagcC	7	1	LCK-1
uggaucuuguuugGcagca	1	1	JT-2
uggaucuuguuugacCgca	1	1	JCK-3
uggaucuuguuugacagcU	1354	1	LT-1
uggaucCuguuugacagca	1	1	LT-2
uggGucuuguuugacagca	1	1	JCK-1
uggCucuuguuugacagca	2	1	LT-1
uAgaucuuguuugacagca	1	1	JT-1
uggaucuuguuugacagcG	2	1	JCK-3
uAgaucuuguuugacagca	1	1	LT-3
uggaucuuguuugCcagca	2	1	LT-3
uggaucuugNuugacagca	1	1	LCK-1
uggaucuCguuugacagca	1	1	LCK-2
uggCucuuguuugacagca	5	1	JT-1
uggaucuuguuugacCgca	2	1	LT-3
uUgaucuuguuugacagca	5	1	JT-2
uggaucuuguuugacCgca	1	1	JT-1
uggaucuuguuugacagca	980	0	JT-2

Mature **Star**

```
uccaucuuuuucguuuacucucuggaucuuguuugacagcacucuccuauauuuuaccauucaucuuuaaaugcgaaauggcgaugaugauggagugcugucaacaugauccagagggugcagucgaauaagauacagagggaccauccacaguaccuacucucaucacaaucuuauucuucuccucacuuauuauuuuucugaucauuuucuuuuacucuauugaugaaguguuauguuuugauucuu
```

......................uNgaucuuguuugacagca	1	1	LCK-3
......................uggCucuuguuugacagca	4	1	LCK-3
......................uggaucuuguuugacagcG	11	1	LCK-3
......................uAgaucuuguuugacagca	1	1	LT-2
......................uggaucuuguuugacCgca	2	1	JCK-2
......................uAgaucuuguuugacagca	3	1	LCK-3
......................uggaucuuguuugacagUa	11	1	JCK-3
......................uggaucuCguuugacagca	1	1	LT-2
......................uggaucuuguuugacagUa	12	1	JCK-1
......................uggaucuuguuugacagUa	11	1	JT-2
......................uggaucuuguuugacagcG	11	1	LT-3
......................uggaucuuguuugacagca	3633	0	LCK-2
......................uggaucuuAuuugacagca	1	1	LCK-2
......................uggaucCuguuugacagca	1	1	LCK-1
......................uggaucuuguuugacCgca	2	1	JT-3
......................ugUaucuuguuugacagca	1	1	LCK-2
......................uggCucuuguuugacagca	2	1	LT-3
......................uggauAuuguuugacagca	1	1	JCK-3
......................uggaucuuguuugacagca	1105	0	JCK-3
......................uggaucuuguuugacagAa	1	1	LT-3
......................uggaucuuguuugacagcC	8	1	LCK-2
......................ugNaucuuguuugacagca	9	1	LT-2
......................uggCucuuguuugacagca	2	1	JCK-1
......................uggaucuuguuugCcagca	11	1	LT-2
......................uggNucuuguuugacagca	1	1	LCK-1
......................uggaucuuguuugacagUa	11	1	LT-3
......................uggaucuuguuugacCgca	3	1	LCK-3
......................ugAaucuuguuugacagca	1	1	JCK-3
......................uggaucGuguuugacagca	1	1	LCK-2
......................ugAaucuuguuugacagca	1	1	JT-1
......................uggaucuuguuugCcagca	5	1	JCK-2
......................uggCucuuguuugacagca	7	1	LCK-2
......................uUgaucuuguuugacagca	5	1	JT-3
......................uggaucuuguuugacagcC	21	1	LT-3
......................uggaucuuguuugacagcG	6	1	JCK-1
......................uggaucuuguuugCcagca	6	1	LCK-2
......................uggaucuuguuugacagcU	1214	1	JCK-2
......................uggCucuuguuugacagca	12	1	LT-2
......................uggCucuuguuugacagca	3	1	JT-2
......................uggaucGuguuugacagca	1	1	JT-3
......................uggaucuuguuugacagcU	1503	1	LCK-2
......................uggaucuuguuugacagcU	279	1	JT-2
......................uUgaucuuguuugacagca	1	1	LT-3
......................uggaucuuguuugacagca	1294	0	JT-3
......................uggaucuuguuugacagUa	24	1	JCK-2
......................uggaucuuguuugacagca	3109	0	LCK-3
......................uggaucuuguuugacagcC	5	1	JCK-2
......................uggaucuuguuugacagcC	3	1	JT-1
......................uNgaucuuguuugacagca	3	1	JT-1
......................uggaucuuguuugCcagca	2	1	JCK-1
......................uggaucuuguuugacCgcac	2	1	JCK-2
......................uggaucuuguuugacagUac	1	1	JCK-3
......................uggaucuuguuugacagcac	249	0	JT-3
......................uggaucuuguuugCcagcac	3	1	JCK-2
......................uggaucuuguuugacagcaA	1	1	JCK-2
......................uggCucuuguuugacagcac	2	1	LT-3
......................uAgaucuuguuugacagcac	4	1	LT-2
......................uggaucuuguuugacagcaA	4	1	JCK-3
......................uggaucuuguuugacagcUc	51	1	LT-3
......................uggaucuuguuugacagcUc	77	1	LT-2
......................uggaucuuguuugacagcUc	15	1	JCK-2
......................uggaucuuguuugacagcac	715	0	LT-3
......................uggaucuuguuugCcagcac	1	1	LT-1
......................uggaCcuuguuugacagcac	1	1	JCK-2
......................uggaucuuguuugacagcaU	66	1	LCK-3
......................uggaucuuCuuugacagcac	1	1	LCK-3
......................uggaucuuguuugacCgcac	3	1	LT-3
......................uggaucuuguuugacagcaU	26	1	JCK-3
......................uggaucuuguuugacagcac	982	0	LT-2
......................uggGucuuguuugacagcac	2	1	LT-2

Mature Star

uccaucuuuuucguuuacucucuggaucuuguuugacagcacucuccuauauuuuaccauucaucuuuaaaugcgaaauggcgaugaugauggagugcugucaacaugauccagagggugcagucgaauaagauacagagggaccauccacaguaccuacucucaucacaaucuuauucuucuccucacuuauuauuuuucugaucauuuucuuuuacucuauugaugaaguguuauguuuugauucuu			
......................uggaucuuguuugacagcUc	36	1	LT-1
......................uggaucuuguuugacagcGc	1	1	LCK-3
......................uggaucuuguuugCcagcac	2	1	JT-1
......................uggaucuuguuugacagcaU	16	1	JT-1
......................uggaucuuguuugacagcaU	26	1	LT-3
......................uggaucuuguuugacagcaA	1	1	LCK-2
......................uggaucuuguuugacagcCc	1	1	JT-1
......................uggaucuuguuugacagcaU	26	1	LT-2
......................uggaucuuguuugacagcaA	3	1	LT-3
......................uggCucuuguuugacagcac	1	1	LCK-1
......................uggaucuuguuugacagcaA	1	1	JT-3
......................Aggaucuuguuugacagcac	1	1	LCK-3
......................uAgaucuuguuugacagcac	1	1	LT-1
......................uggaucuuguuugacagcac	666	0	LT-1
......................uggaucuuguuugacagcUc	6	1	JCK-1
......................uggaucuuguuugCcagcac	1	1	JCK-1
......................uNgaucuuguuugacagcac	1	1	JCK-1
......................uggaucuuguuugacagcUc	31	1	LCK-2
......................uggaucuuguuugGcagcac	1	1	LT-3
......................uggCucuuguuugacagcac	1	1	JCK-1
......................uggaucuuguuugCcagcac	1	1	JCK-3
......................uggCucuuguuugacagcac	2	1	JT-3
......................uggaucuugCuugacagcac	1	1	LT-2
......................uggaCcuuguuugacagcac	1	1	JCK-3
......................uggaucuuguuugacagcaU	22	1	LT-1
......................uggaCcuuguuugacagcac	1	1	JT-1
......................uAgaucuuguuugacagcac	1	1	LCK-1
......................uggaucuuguuuAacagcac	1	1	LCK-2
......................uggaucuuguuugNcagcac	2	1	LCK-1
......................uggaucuuguuNgacagcac	1	1	JT-2
......................uggaucuuguuugacagcac	525	0	LCK-2
......................uggaucuuguuugacagcac	1318	0	LCK-3
......................uggaucuuguuugGcagcac	1	1	LCK-1
......................uggaucuuguuugacagcCc	2	1	LT-1
......................uggaucuuguuugacagcac	216	0	JT-1
......................uggaucuuguuugCcagcac	4	1	LT-2
......................uggaucuuguuugacagcac	586	0	JCK-1
......................uggaucuuguuugacGgcac	1	1	LT-2
......................uggCucuuguuugacagcac	9	1	LT-2
......................uggaucuuguCugacagcac	1	1	JCK-2
......................uggaucuuguuugCcagcac	1	1	JT-2
......................uggaucuuguuugacagcCc	1	1	LT-2
......................uggCucuuguuugacagcac	1	1	LCK-2
......................uNgaucuuguuugacagcac	1	1	LCK-3
......................uggaucuuguuugacagcac	170	0	JT-2
......................uggCucuuguuugacagcac	1	1	LT-1
......................uggauNuuguuugacagcac	1	1	LCK-1
......................uggaucuuguuugacagcaA	3	1	JCK-1
......................uggCucuuguuugacagcac	5	1	LCK-3
......................uggaucuuguuugaNagcac	2	1	LCK-1
......................uggaucuuguuugCcagcac	1	1	LCK-3
......................uggaucuuguuugacagcac	899	0	JCK-2
......................uggaucuuguuugacagcNc	1	1	JCK-1
......................uggaucuuguuugacagcaN	2	1	JCK-1
......................uggaucuuguuugacagcac	687	0	JCK-3
......................uggaucuuguuugacagcUc	26	1	LCK-1
......................uggaucuuguuugacagcUc	63	1	LCK-3
......................uggaucuuUuuugacagcac	1	1	LT-3
......................uAgaucuuguuugacagcac	1	1	LCK-3
......................uggaucuuguuugacagcUc	7	1	JCK-3
......................uggaucuuguuugacagcaU	46	1	JCK-2
......................ugNaucuuguuugacagcac	3	1	LT-2
......................uggaucuuguuugacagcaU	8	1	JCK-1
......................uggaucuuguuugacagcaG	1	1	LT-1
......................uggCucuuguuugacagcac	2	1	JCK-2
......................uggaucuuguuugCcagcac	1	1	LT-3
......................uggaucuuguuugacagcac	545	0	LCK-1
......................uggaucuuguuugacCgcac	1	1	LT-2
......................ugAaucuuguuugacagcac	1	1	JCK-1
......................uggaucuuguuugacagcaUu	21	1	JT-3

```
                      Mature                                                                    Star
uccaucuuuuucguuuacucucuggaucuuguuugacagcacucuccuauauuuuaccauucaucuuuaaaugcgaaauggcgaugaugaugggagugcugucaacaugauccagagggugcagucgaauaagauacagagggaccauccacaguaccuacucucaucacaaucuuauucuucuccucacuuauuauuuuucugaucauuuucuuuuacucuauugaugaaguguuauguuuugauucuu
......................uggaucuuguuugacagGacu..............................................................................................................................................................................................................	2	1	JCK-2
......................uggaucuuguuugacagcaAu..............................................................................................................................................................................................................	1	1	LCK-3
......................uggaucuuguuugacagcaUu..............................................................................................................................................................................................................	55	1	JT-1
......................uggaucuuguuugacCgcacu..............................................................................................................................................................................................................	11	1	LT-3
......................uggaucuuguuugacCgcacu..............................................................................................................................................................................................................	19	1	JCK-1
......................uggaucuuguuugacagcacu..............................................................................................................................................................................................................	979	0	JT-3
......................uggaucuuguuugacagcGcu..............................................................................................................................................................................................................	2	1	JCK-2
......................uggaucuuguuNgacagcacu..............................................................................................................................................................................................................	1	1	JCK-2
......................uggaucuuguuugacagcUcu..............................................................................................................................................................................................................	1	1	LT-3
......................uggaucCuguuugacagcacu..............................................................................................................................................................................................................	2	1	LT-2
......................uggaucuugCuugacagcacu..............................................................................................................................................................................................................	4	1	LCK-3
......................uggaucuuguuAgacagcacu..............................................................................................................................................................................................................	1	1	JCK-1
......................Cggaucuuguuugacagcacu..............................................................................................................................................................................................................	4	1	LT-2
......................uggaucuuguuugacagcCcu..............................................................................................................................................................................................................	34	1	LT-2
......................uggGucuuguuugacagcacu..............................................................................................................................................................................................................	3	1	LT-3
......................uggauNuuguuugacagcacu..............................................................................................................................................................................................................	3	1	LCK-1
......................ugCaucuuguuugacagcacu..............................................................................................................................................................................................................	1	1	LCK-3
......................uggaucuuguuugaAagcacu..............................................................................................................................................................................................................	4	1	JCK-2
......................uggaucuuguuuAacagcacu..............................................................................................................................................................................................................	3	1	LT-1
......................uggaucuuguuugacagcaAu..............................................................................................................................................................................................................	5	1	JT-2
......................uggaucuuAuuugacagcacu..............................................................................................................................................................................................................	6	1	LT-1
......................uUgaucuuguuugacagcacu..............................................................................................................................................................................................................	2	1	LCK-3
......................uUgaucuuguuugacagcacu..............................................................................................................................................................................................................	10	1	JCK-3
......................uNgaucuuguuugacagcacu..............................................................................................................................................................................................................	104	1	JCK-2
......................uggaucuuAuuugacagcacu..............................................................................................................................................................................................................	20	1	JCK-2
......................uggaucuuguuugacagcacG..............................................................................................................................................................................................................	12	1	LT-3
......................uNgaucuuguuugacagcacu..............................................................................................................................................................................................................	4	1	JT-1
......................uggaucuugGuugacagcacu..............................................................................................................................................................................................................	1	1	JCK-2
......................uggaucuuguuugacagGacu..............................................................................................................................................................................................................	24	1	JCK-1
......................uggaucuuguCugacagcacu..............................................................................................................................................................................................................	5	1	JT-2
......................Cggaucuuguuugacagcacu..............................................................................................................................................................................................................	7	1	JCK-3
......................uggaucuuguuugacCgcacu..............................................................................................................................................................................................................	26	1	LT-2
......................uggaucuuguuugacaCcacu..............................................................................................................................................................................................................	1	1	LT-3
......................uNgaucuuguuugacagcacu..............................................................................................................................................................................................................	2	1	LT-3
......................uggaucuuguuugGcagcacu..............................................................................................................................................................................................................	2	1	LT-1
......................uggaucuuCuuugacagcacu..............................................................................................................................................................................................................	1	1	JCK-1
......................Cggaucuuguuugacagcacu..............................................................................................................................................................................................................	1	1	JT-1
......................uggaucuuguuugGcagcacu..............................................................................................................................................................................................................	3	1	JCK-2
......................uggaucuuguuugacagcacu..............................................................................................................................................................................................................	27651	0	LT-3
......................uCgaucuuguuugacagcacu..............................................................................................................................................................................................................	1	1	LCK-3
......................uggauGuuguuugacagcacu..............................................................................................................................................................................................................	1	1	JCK-3
......................uggaucuuguuugacagcaUu..............................................................................................................................................................................................................	1426	1	JCK-1
......................ugUaucuuguuugacagcacu..............................................................................................................................................................................................................	1	1	JT-1
......................uggaucuuguuugaAagcacu..............................................................................................................................................................................................................	3	1	LT-1
......................uggaucuuguuugacagcCcu..............................................................................................................................................................................................................	4	1	LCK-1
......................uggaucuuguuugacagcacu..............................................................................................................................................................................................................	4848	0	LCK-1
......................uggaucuuguuugacagcacG..............................................................................................................................................................................................................	3	1	LT-2
......................uggaucuuguuugacGgcacu..............................................................................................................................................................................................................	1	1	JT-3
......................uAgaucuuguuugacagcacu..............................................................................................................................................................................................................	30	1	LT-2
......................uggauAuuguuugacagcacu..............................................................................................................................................................................................................	2	1	JCK-2
......................uggaucuuUuuugacagcacu..............................................................................................................................................................................................................	1	1	LCK-3
......................uggaucuCguuugacagcacu..............................................................................................................................................................................................................	4	1	JCK-3
......................uggaucuuguuugacagcaUu..............................................................................................................................................................................................................	68	1	LT-2
......................uggaucuuguuugacagcaUu..............................................................................................................................................................................................................	64	1	LT-3
......................uggaucuCguuugacagcacu..............................................................................................................................................................................................................	1	1	LT-1
......................uAgaucuuguuugacagcacu..............................................................................................................................................................................................................	13	1	LCK-3
......................uggaucuCguuugacagcacu..............................................................................................................................................................................................................	1	1	LCK-1
......................uggaucuuguuNgacagcacu..............................................................................................................................................................................................................	1	1	JT-1
......................uNgaucuuguuugacagcacu..............................................................................................................................................................................................................	1	1	LCK-1
......................uggaCcuuguuugacagcacu..............................................................................................................................................................................................................	2	1	LT-2
......................uggaucuuAuuugacagcacu..............................................................................................................................................................................................................	1	1	LCK-2
......................uggaucCuguuugacagcacu..............................................................................................................................................................................................................	10	1	JCK-1
......................uggCucuuguuugacagcacu..............................................................................................................................................................................................................	9	1	LCK-2
......................uggaucuugAuugacagcacu..............................................................................................................................................................................................................	1	1	JCK-2
......................uggaucuCguuugacagcacu..............................................................................................................................................................................................................	5	1	JCK-1
......................uggaucuuguuugacagcaUu..............................................................................................................................................................................................................	37	1	LCK-2
......................uggaucuCguuugacagcacu..............................................................................................................................................................................................................	3	1	LCK-3
......................ugUaucuuguuugacagcacu..............................................................................................................................................................................................................	1	1	LCK-2
......................uggauUuuguuugacagcacu..............................................................................................................................................................................................................	1	1	LCK-3
......................uggaucuuguuugGcagcacu..............................................................................................................................................................................................................	1	1	LT-2
```

Mature Star

uccaucuuuuucguuuacucucuggaucuuguuugacagcacucuccuauauuuuaccauucaucuuuaaaugcgaaauggcgaugaugaugggagugcugucaacaugauccagagggugcagucgaauaagauacagagggaccauccacaguaccuacucucaucacaaucuuauucuucuccucacuuauuauuuuucugaucauuuucuuuuacucuauugaugaaguguuauguuuugauucuu			
......................uggaucuuguuugacGgcacu	10	1	JCK-2
......................uggaucuuAuuugacagcacu	6	1	JCK-1
......................uggaucuuguuugacagcaAu	3	1	JCK-1
......................uggaucuuguuugacagcacG	1	1	JT-2
......................uggaucuuguuuAacagcacu	2	1	LT-2
......................uAgaucuuguuugacagcacu	22	1	LT-3
......................uAgaucuuguuugacagcacu	4	1	LCK-2
......................Cggaucuuguuugacagcacu	9	1	LT-3
......................uggaCcuuguuugacagcacu	1	1	LCK-2
......................uggaucuuguuugacagcGcu	2	1	JCK-1
......................uggaucCuguuugacagcacu	2	1	LT-3
......................uggaucuuguuugacagGacu	2	1	JCK-3
......................uggaucuuguuugacGgcacu	2	1	LT-1
......................uUgaucuuguuugacagcacu	1	1	JT-3
......................uAgaucuuguuugacagcacu	8	1	JCK-2
......................uggNucuuguuugacagcacu	3	1	JCK-3
......................uggCucuuguuugacagcacu	2	1	JT-2
......................uggGucuuguuugacagcacu	5	1	JCK-3
......................ugAaucuuguuugacagcacu	19	1	JCK-2
......................ugCaucuuguuugacagcacu	2	1	LT-2
......................uggaucuugGuugacagcacu	1	1	JCK-3
......................uggaucuuguuugacagcaAu	1	1	LT-1
......................uggaucuuguuugacagUacu	18	1	JCK-1
......................uggaucuuguuugacagcacG	16	1	JCK-2
......................uggaucuuguuugCcagcacu	105	1	JCK-3
......................uggaucuuguuAgacagcacu	1	1	JCK-2
......................uggaucuuguuugCcagcacu	86	1	JCK-1
......................uggaCcuuguuugacagcacu	13	1	JCK-3
......................uggaucuuguuuUacagcacu	4	1	LT-1
......................ugAaucuuguuugacagcacu	3	1	LCK-2
......................uggaucuuguuugNcagcacu	15	1	LCK-1
......................uggaCcuuguuugacagcacu	14	1	JCK-2
......................uggaucuugCuugacagcacu	6	1	JCK-3
......................uggaucGuguuugacagcacu	2	1	JCK-1
......................uCgaucuuguuugacagcacu	1	1	JCK-3
......................uggaCcuuguuugacagcacu	3	1	LT-1
......................ugUaucuuguuugacagcacu	2	1	LT-2
......................uggaucuuAuuugacagcacu	4	1	JCK-3
......................uggaucuuguuAgacagcacu	1	1	LCK-3
......................uggaucuuguuugacCgcacu	3	1	JT-1
......................uggaucuuguuugacagcacu	20839	0	LT-2
......................uggaucuuguCugacagcacu	1	1	LT-2
......................uggaucuuguuugacagAacu	1	1	JCK-1
......................uggaucuuguuugacaCcacu	3	1	JCK-2
......................uggNucuuguuugacagcacu	22	1	JCK-1
......................uggaucuuguCugacagcacu	6	1	JCK-1
......................uggaucuuguuugacGgcacu	9	1	JCK-3
......................uggGucuuguuugacagcacu	8	1	JCK-2
......................uggaucuuguuugacagcacu	66730	0	JCK-2
......................uggaucuuguuugacagcGcu	1	1	JT-3
......................uggaucuuguuugacagcUcu	6	1	LT-2
......................uggaucuuAuuugacagcacu	4	1	LCK-3
......................uggaucuugGuugacagcacu	1	1	JCK-1
......................uggCucuuguuugacagcacu	65	1	LT-1
......................Gggaucuuguuugacagcacu	1	1	JCK-2
......................uggaucuuguuugacagcCcu	31	1	LT-3
......................uggaucuuguuugacaCcacu	5	1	JCK-3
......................uggaucuuUuuugacagcacu	1	1	LT-3
......................uggaucuuAuuugacagcacu	1	1	LCK-1
......................uggaucuuguuugaNagcacu	17	1	LCK-1
......................uggaucuuguuugCcagcacu	159	1	JCK-2
......................uggaucuuguuuAacagcacu	6	1	JCK-1
......................uggaucGuguuugacagcacu	1	1	LT-1
......................uggaucuuguuuAacagcacu	1	1	JT-1
......................uAgaucuuguuugacagcacu	15	1	JCK-3
......................ugAaucuuguuugacagcacu	11	1	LT-3
......................uggaucuuguCugacagcacu	1	1	LT-3
......................uggaucuuguuugGcagcacu	3	1	JCK-3
......................uggaucuuNuuugacagcacu	1	1	JCK-3
......................uggaucuuguuuAacagcacu	1	1	LT-3

Mature **Star**

uccaucuuuuucguuuacucucuggaucuuguuugacagcacucuccuauauuuuaccauucaucuuuaaaugcgaaauggcgaugaugauggggagugcugucaacaugauccagagggugcagucgaauaagauacagagggaccauccacaguaccuacucucaucacaaucuuauucuucuccucacuuauuauuuuucugaucauuuucuuuuacucuauugaugaaguguuauguuuugauucuu			
......................uggaucuuguuugaAagcacu........	2	1	JCK-1
......................ugUaucuuguuugacagcacu........	3	1	LT-3
......................uggaucuuguuugUcagcacu........	2	1	JCK-2
......................uggaucuuguuugacagcaAu........	3	1	LT-3
......................uggauUuuguuugacagcacu........	11	1	JCK-2
......................uNgaucuuguuugacagcacu........	66	1	JCK-1
......................uggaucuuguuugacagcacG........	10	1	JCK-1
......................uggaucuugCuugacagcacu........	6	1	JCK-2
......................uggaucuuUuuugacagcacu........	3	1	JCK-1
......................uggaucuuguuugacCgcacu........	5	1	LCK-2
......................uggaucuuguuugacGgcacu........	3	1	LT-3
......................uggaucuuguuugacagcacu........	658	0	JT-2
......................uggauUuuguuugacagcacu........	1	1	LT-2
......................uUgaucuuguuugacagcacu........	2	1	LT-1
......................uggauNuuguuugacagcacu........	1	1	JCK-2
......................uggCucuuguuugacagcacu........	118	1	JCK-3
......................uggaucuuguuugacagcNcu........	22	1	JCK-1
......................uggaucuuguuugacagcacN........	22	1	JCK-1
......................uggaucuuCuuugacagcacu........	1	1	JCK-3
......................uggaucuuguuGgacagcacu........	1	1	JCK-2
......................Cggaucuuguuugacagcacu........	1	1	JT-3
......................uggaucuuguuugGcagcacu........	5	1	LCK-1
......................uggaucuuguuugacagcaUu........	2361	1	JCK-2
......................uggaucuuguuugCcagcacu........	2	1	JT-2
......................uUgaucuuguuugacagcacu........	4	1	JCK-2
......................uggaucCuguuugacagcacu........	12	1	JCK-2
......................uggauUuuguuugacagcacu........	3	1	JCK-1
......................Cggaucuuguuugacagcacu........	3	1	LT-1
......................ugAaucuuguuugacagcacu........	4	1	LCK-3
......................uggaucuuguuugacagcacu........	38814	0	JCK-1
......................uggaucuuguuugacagcaGu........	2	1	JCK-1
......................uggaucuuguuugacagcaAu........	10	1	JCK-2
......................uggaucuuguuugacagcaNu........	42	1	JCK-1
......................uggCucuuguuugacagcacu........	1	1	JT-3
......................uggGucuuguuugacagcacu........	1	1	JT-1
......................uggaucuuguuugCcagcacu........	5	1	JT-1
......................uggCucuuguuugacagcacu........	7	1	JT-1
......................uggCucuuguuugacagcacu........	200	1	LT-2
......................uggaucuuguuuAacagcacu........	1	1	LCK-1
......................uggauUuuguuugacagcacu........	2	1	LT-3
......................ugUaucuuguuugacagcacu........	4	1	JCK-3
......................uggaucuGguuugacagcacu........	1	1	JCK-2
......................uggaucuuguGugacagcacu........	15	1	JCK-1
......................uggGucuuguuugacagcacu........	8	1	JCK-1
......................uggaucuuguuuNacagcacu........	1	1	LCK-1
......................uggCucuuguuugacagcacu........	92	1	JCK-1
......................uggaucuuUuuugacagcacu........	4	1	JCK-2
......................uggaucuuguuugacagcCcu........	17	1	JCK-3
......................uggaucuuguuugacagcacG........	7	1	LCK-3
......................uggaucuuUuuugacagcacu........	3	1	LT-2
......................uggaucuuguuugacagAacu........	1	1	JCK-2
......................uggaGcuuguuugacagcacu........	2	1	JCK-3
......................uggCucuuguuugacagcacu........	8	1	LCK-1
......................uggaucuuguuugacagcNcu........	1	1	LCK-1
......................uggaucuuguuugacCgcacu........	29	1	JCK-2
......................uggaucuuguuugacagcCcu........	4	1	LCK-2
......................uNgaucuuguuugacagcacu........	1	1	LCK-2
......................uggaucuuguuugGcagcacu........	1	1	LT-3
......................uggaucuuguuugacagUacu........	11	1	LT-2
......................uggaucuuguuGgacagcacu........	10	1	JCK-1
......................ugAaucuuguuugacagcacu........	2	1	LCK-1
......................uggaucuuguuugacagUacu........	13	1	JCK-3
......................uggNucuuguuugacagcacu........	1	1	LCK-1
......................uggaucuuguuugacagUacu........	34	1	JCK-2
......................uggaucuuguuugaGagcacu........	1	1	JT-1
......................uggaucuuguuugacaCcacu........	1	1	JT-1
......................uggaucuuguuugacagcCcu........	16	1	LCK-3
......................uggaucuuguuugacagcaUu........	22	1	JT-2
......................uggaucuuguuugacagcaUu........	1732	1	JCK-3
......................uggaucuuguuugacagcacG........	10	1	LT-1

```
                      Mature                                                                                 Star
uccaucuuuuucguuuacucucuggaucuuguuugacagcacucuccuauauuuuaccauucaucuuuaaaugcgaaauggcgaugaugaugggagugcugucaacaugauccagagggugcagucgaauaagauacagagggaccauccacaguaccuacucucaucacaaucuuauucuucuccucacuuauuauuuuucugaucauuuucuuuuacucuauugaugaaguguuauguuuugauucuu
......................uggaucuuguuugCcagcacu..............................................................................................................................................................................................  67	1	LT-3
......................uggaucuuguuugacagcCcu..............................................................................................................................................................................................  53	1	JCK-1
......................uggaucuugCuugacagcacu..............................................................................................................................................................................................  2	1	JCK-1
......................ugCaucuuguuugacagcacu..............................................................................................................................................................................................  1	1	JCK-3
......................uggaucuCguuugacagcacu..............................................................................................................................................................................................  10	1	JCK-2
......................uggaucuuguuugacagcaAu..............................................................................................................................................................................................  3	1	JCK-3
......................uggaucuuguGugacagcacu..............................................................................................................................................................................................  2	1	LT-2
......................uggaucuuguuuUacagcacu..............................................................................................................................................................................................  6	1	JCK-2
......................uggaucuuguuugacagcUcu..............................................................................................................................................................................................  1	1	JCK-2
......................uggaucuuguuugacagcacu..............................................................................................................................................................................................  5029	0	LCK-2
......................uggaucuuAuuugacagcacu..............................................................................................................................................................................................  9	1	LT-2
......................uggaucuuguCugacagcacu..............................................................................................................................................................................................  7	1	JCK-3
......................uggauAuuguuugacagcacu..............................................................................................................................................................................................  1	1	JCK-3
......................uggaucuuguuugacagcaAu..............................................................................................................................................................................................  1	1	LCK-2
......................uggaucuuAuuugacagcacu..............................................................................................................................................................................................  3	1	LT-3
......................uggaucuuguuugacagcaAu..............................................................................................................................................................................................  4	1	LT-2
......................uggaucuuguuugacagcGcu..............................................................................................................................................................................................  3	1	LT-2
......................uggaucuuguuugacCgcacu..............................................................................................................................................................................................  3	1	LCK-1
......................uggaucuuguuugCcagcacu..............................................................................................................................................................................................  80	1	LT-2
......................uggNucuuguuugacagcacu..............................................................................................................................................................................................  1	1	JCK-2
......................uggaucuuguuugCcagcacu..............................................................................................................................................................................................  1	1	JT-3
......................uggaucuuguuugCcagcacu..............................................................................................................................................................................................  61	1	LT-1
......................uggaucuuguuugacagcacu..............................................................................................................................................................................................  20263	0	LCK-3
......................uggaucuuguuuAacagcacu..............................................................................................................................................................................................  9	1	JCK-2
......................uggCucuuguuugacagcacu..............................................................................................................................................................................................  152	1	JCK-2
......................uggaCcuuguuugacagcacu..............................................................................................................................................................................................  4	1	LT-3
......................uggaucuuguuugacCgcacu..............................................................................................................................................................................................  28	1	JCK-3
......................uggaucuuguuugacagUacu..............................................................................................................................................................................................  1	1	JT-2
......................uggaucuuguuuAacagcacu..............................................................................................................................................................................................  1	1	LCK-3
......................uggaucuuguuugacagcUcu..............................................................................................................................................................................................  5	1	LT-1
......................uggaucuuguuugacagcaAu..............................................................................................................................................................................................  4	1	JT-3
......................ugUaucuuguuugacagcacu..............................................................................................................................................................................................  2	1	JCK-1
......................uUgaucuuguuugacagcacu..............................................................................................................................................................................................  7	1	LT-2
......................uggCucuuguuugacagcacu..............................................................................................................................................................................................  95	1	LT-3
......................uggaucCuguuugacagcacu..............................................................................................................................................................................................  8	1	JCK-3
......................uggaucuuguCugacagcacu..............................................................................................................................................................................................  3	1	LT-1
......................uggaucuugCuugacagcacu..............................................................................................................................................................................................  4	1	LT-2
......................uggaucCuguuugacagcacu..............................................................................................................................................................................................  1	1	LCK-1
......................uggaucuuguuuUacagcacu..............................................................................................................................................................................................  6	1	JCK-1
......................uNgaucuuguuugacagcacu..............................................................................................................................................................................................  55	1	JCK-3
......................uAgaucuuguuugacagcacu..............................................................................................................................................................................................  14	1	LT-1
......................uggauUuuguuugacagcacu..............................................................................................................................................................................................  4	1	JCK-3
......................uggaucuuguuugacagcaUu..............................................................................................................................................................................................  102	1	LCK-3
......................uggaucuuguuuCacagcacu..............................................................................................................................................................................................  1	1	JCK-3
......................uggaucuugCuugacagcacu..............................................................................................................................................................................................  1	1	LT-3
......................ugAaucuuguuugacagcacu..............................................................................................................................................................................................  6	1	LT-1
......................ugAaucuuguuugacagcacu..............................................................................................................................................................................................  8	1	JCK-3
......................uggaucuuguuNgacagcacu..............................................................................................................................................................................................  121	1	JCK-1
......................uggaucuuguuugaGagcacu..............................................................................................................................................................................................  8	1	LCK-1
......................uggaucuuguuGgacagcacu..............................................................................................................................................................................................  1	1	JCK-3
......................uggaucuuguuugacagNacu..............................................................................................................................................................................................  3	1	JCK-3
......................ugAaucuuguuugacagcacu..............................................................................................................................................................................................  3	1	JCK-1
......................uggaucuCguuugacagcacu..............................................................................................................................................................................................  3	1	LT-3
......................uggaucuuguuugCcagcacu..............................................................................................................................................................................................  7	1	LCK-2
......................uggaucCuguuugacagcacu..............................................................................................................................................................................................  4	1	LT-1
......................uNgaucuuguuugacagcacu..............................................................................................................................................................................................  3	1	LT-1
......................uggaucuuguuugacagcacu..............................................................................................................................................................................................  1709	0	JT-1
......................uggaucuuguuugacGgcacu..............................................................................................................................................................................................  7	1	JCK-1
......................uggaucuuguuugacCgcacu..............................................................................................................................................................................................  7	1	LCK-3
......................uggaucuuguuugacagcacu..............................................................................................................................................................................................  48441	0	JCK-3
......................uggaucuuguuugacagcCcu..............................................................................................................................................................................................  2	1	JT-1
......................uggaucuuguuuCacagcacu..............................................................................................................................................................................................  1	1	JCK-2
......................uggaucuuguuugacGgcacu..............................................................................................................................................................................................  7	1	LT-2
......................uggaCcuuguuugacagcacu..............................................................................................................................................................................................  1	1	LCK-3
......................uggaucuuguuugaAagcacu..............................................................................................................................................................................................  5	1	JCK-3
......................uggaucuuguuugacCgcacu..............................................................................................................................................................................................  12	1	LT-1
......................uggaucuuguuuUacagcacu..............................................................................................................................................................................................  2	1	LT-2
......................uggaucuuguuuUacagcacu..............................................................................................................................................................................................  2	1	JCK-3
......................uggaucuuguuuCacagcacu..............................................................................................................................................................................................  2	1	JCK-1
......................ugNaucuuguuugacagcacu..............................................................................................................................................................................................  1	1	LCK-1
```

Mature **Star**

uccaucuuuuucguuuacucucuggaucuuguuugacagcacucuccuauauuuuaccauucaucuuuaaaugcgaaauggcgaugaugaugggagugcugucaacaugauccagagggugcagucgaauaagauacagagggaccauccacaguaccuacucucaucacaaucuuauucuucucccucacuuauuauuuuucugaucauuuucuuuuacucuauugaugaaguguuauguuuugauucuu

uggaucuuguuugacagcCcu	74	1	JCK-2
uggaucuuguuugacagcaUu	20	1	LT-1
uggaucuuCuuugacagcacu	1	1	JCK-2
uAgaucuuguuugacagcacu	5	1	JCK-1
uggaucuuguuugacagcacG	2	1	LCK-1
ugUaucuuguuugacagcacu	1	1	JCK-2
uggaucuuguuuUacagcacu	3	1	LT-3
uggaucuuguuugacagcCcu	29	1	LT-1
uggaucuuguuugacagUacu	5	1	LT-3
uggaucuuguuugacagcacu	23834	0	LT-1
Cggaucuuguuugacagcacu	6	1	JCK-1
uggaucuuguCugacagcacu	1	1	LCK-2
uggaucuuguuugacaCcacu	5	1	JCK-1
uggCucuuguuugacagcacu	39	1	LCK-3
uggaucuuguuugCcagcacu	5	1	LCK-1
uggaucuuUuuugacagcacu	6	1	JCK-3
uggaCcuuguuugacagcacu	4	1	JCK-1
uggaucuuUuuugacagcacu	2	1	LT-1
uggaucuuguuugacGgcacu	2	1	LCK-3
uggaucuuguuugGcagcacu	3	1	JCK-1
uggaucuuguuugacagAacu	3	1	LT-1
uggaucuuguuugCcagcacu	36	1	LCK-3
uggaucuugCuugacagcacu	2	1	LT-1
uggaucuuguuGgacagcacu	1	1	LT-3
ugNaucuuguuugacagcacu	65	1	LT-2
Cggaucuuguuugacagcacu	11	1	JCK-2
uggaucuuguuugacagcaUu	36	1	LCK-1
ugAaucuuguuugacagcacu	10	1	LT-2
uggaucuuguuuAacagcacu	7	1	JCK-3
uggaucuuguuugacagcacG	13	1	JCK-3
uggaucuuguCugacagcacu	6	1	JCK-2
uUgaucuuguuugacagcacuc	1	1	JCK-2
uggaucuuguuugacagcacuA	59	1	JCK-2
uggauAuuguuugacagcacuc	1	1	JCK-1
uggaucuuguuugacagcacuc	138	0	JT-2
Cggaucuuguuugacagcacuc	1	1	LT-1
ugUaucuuguuugacagcacuc	3	1	JCK-3
uggaucuuUuuugacagcacuc	2	1	JCK-2
ugAaucuuguuugacagcacuc	2	1	JCK-1
uggaucuuguuugacagcCcuc	1	1	LCK-1
uggaucuuguuugacagcacGc	1	1	JCK-2
uggCucuuguuugacagcacuc	1	1	JT-1
uggaucuuguuugNcagcacuc	1	1	LCK-1
uggaucuuguuugacagcacuA	34	1	LT-3
uggaucuuguuugCcagcacuc	19	1	JCK-3
uggaucuuguuugCcagcacuc	24	1	JCK-1
uggaucuuguuugacagcaAuc	1	1	JCK-2
uggaGcuuguuugacagcacuc	1	1	JCK-3
uggaucuuguuugacagcacuG	4	1	JCK-2
uggaucuuguuugacagcacuc	17179	0	JCK-2
uggaucuuguuugacagcaUuc	7	1	JCK-1
ugUaucuuguuugacagcacuc	1	1	JCK-2
Cggaucuuguuugacagcacuc	5	1	JCK-3
uggaucuuguuugacagcCcuc	7	1	JCK-2
uggaucuuguuugacagcacuc	70	0	LCK-2
uggaucuuguuuUacagcacuc	1	1	JCK-3
uggaucuugGuugacagcacuc	3	1	JCK-3
uggCucuuguuugacagcacuc	36	1	JCK-2
uggaucuuguuugacCgcacuc	2	1	LCK-3
uggaucuuguuugacagGacuc	7	1	JCK-1
uggaucuuguuugacagcacuU	97	1	JCK-2
uggCucuuguuugacagcacuc	1	1	JT-2
uggaucuuguuuAacagcacuc	3	1	JCK-2
uggaucuuguuugacagcacuG	11	1	JCK-1
ugAaucuuguuugacagcacuc	2	1	JCK-3
uggNucuuguuugacagcacuc	7	1	JCK-1
uggaucuuguuugacagcacuc	220	0	JT-1
uggaucuuguuugacagUacuc	5	1	JCK-2
ugUaucuuguuugacagcacuc	1	1	JCK-1
uggaCcuuguuugacagcacuc	9	1	JCK-3

Mature **Star**

uccaucuuuuucguuuacucucuggaucuuguuugacagcacucuccuauauuuuaccauucaucuuuaaaugcgaaauggcgaugaugaugggagugcugucaacaugauccagagggugcagucgaauaagauacagagggaccauccacaguaccuacucucaucacaaucuuauucuucuccucacuuauuauuuuucugaucauuuucuuuuacucuauugaugaagguguuauguuuugauucuu

uggCucuuguuugacagcacuc	36	1	JCK-1
uggaucuuguuugacCgcacuc	3	1	JCK-3
uggaucuuguuugacagcacuc	627	0	LT-2
uggaucuuguuugacagcacuG	7	1	LT-1
uggaucuuguuugacagcNcuc	7	1	JCK-1
uggaucuuguuugacagcCcuc	7	1	JCK-1
uggaucuuguuugCcagcacuc	2	1	LT-2
uggGucuuguuugacagcacuc	4	1	JCK-2
uggaCcuuguuugacagcacuc	1	1	LT-2
uggaucuuAuuugacagcacuc	4	1	JCK-2
uggaucuuguuugacagcCcuc	1	1	JT-3
uggaucuuguuugacagcacuc	76	0	LCK-1
uggaucuCguuugacagcacuc	2	1	JCK-3
uNgaucuuguuugacagcacuc	26	1	JCK-1
uggaCcuuguuugacagcacuc	6	1	JCK-1
uggaucuuguuugGcagcacuc	1	1	JCK-1
uggCucuuguuugacagcacuc	3	1	LCK-3
uNgaucuuguuugacagcacuc	15	1	JCK-3
uggaucuuNuuugacagcacuc	1	1	JCK-3
ugAaucuuguuugacagcacuc	2	1	LT-3
uAgaucuuguuugacagcacuc	2	1	LT-1
uggaucuuguGugacagcacuc	5	1	JCK-1
uggaucuuguuugCcagcacuc	44	1	JCK-2
uggaucuuguuugacagcacuG	3	1	LT-3
uggaucuuguuugCcagcacuc	3	1	LCK-3
uggaucuuguuugacagcacuc	520	0	LCK-3
uggaucuuguuugacagcacuA	12	1	LCK-3
uggaucuuguuugacCgcacuc	1	1	LT-1
uggaucuuguuugacagcUcuc	2	1	JCK-2
uggaucuuguuugacaAcacuc	4	1	JCK-1
uggaucuuguuugacagcacuA	32	1	LT-1
uggaucuuguuugCcagcacuc	1	1	JT-2
Cggaucuuguuugacagcacuc	1	1	LT-2
uggaucuuguuugacagcacuU	85	1	JCK-3
uggaucuuguuugacagcacuc	9941	0	JCK-1
uggaucuuguuugacGgcacuc	2	1	JCK-3
uggaucuuUuuugacagcacuc	1	1	JCK-3
uggauUuuguuugacagcacuc	7	1	JCK-2
uggaucuuguuugacaCcacuc	1	1	JCK-3
uggaucuuguuugacagcGcuc	1	1	JCK-1
ugAaucuuguuugacagcacuc	1	1	LT-1
uggaucuuguuugacagcacuU	48	1	LT-3
uggaucuuAuuugacagcacuc	1	1	LT-2
uggaucuuguuugacaAcacuc	3	1	JCK-2
uggCucuuguuugacagcacuc	33	1	JCK-3
uggaucuCguuugacagcacuc	2	1	JCK-1
uggaucuuguuugacagcacuN	9	1	JCK-1
uggaucuuguuugacagcaNuc	11	1	JCK-1
uggGucuuguuugacagcacuc	1	1	JCK-3
uUgaucuuguuugacagcacuc	1	1	JCK-1
uggaucuuguuugacGgcacuc	1	1	LT-1
uggaucuuguuugacagcacuU	4	1	JT-3
uggCucuuguuugacagcacuc	4	1	LT-2
uggaucuuguuugCcagcacuc	7	1	LT-1
uAgaucuuguuugacagcacuc	1	1	LT-3
uggaucuuguuugacagcacuU	22	1	LCK-2
Cggaucuuguuugacagcacuc	2	1	JCK-1
Cggaucuuguuugacagcacuc	1	1	LCK-3
uggaucuuguuNgacagcacuc	29	1	JCK-1
uAgaucuuguuugacagcacuc	3	1	JCK-1
uAgaucuuguuugacagcacuc	10	1	JCK-3
uggaucuuguuugacagcacuc	1262	0	LT-1
uAgaucuuguuugacagcacuc	1	1	LT-2
uUgaucuuguuugacagcacuc	1	1	JCK-3
uAgaucuuguuugacagcacuc	7	1	JCK-2
uggaucuuguuugacagcUcuc	1	1	JCK-1
uggaucuuguuugacagcacuA	33	1	LT-2
uggaucuuguuugacaAcacuc	6	1	JCK-3
uggaucuuguuugacagcacuU	27	1	LT-2
uggaucuuguuuAacagcacuc	1	1	JCK-3

```
                            Mature                                                                  Star
uccaucuuuuucguuuacucucuggaucuuguuugacagcacucuccuauauuuuaccauucaucuuuaaaugcgaaauggcgaugaugauggagugcugucaacaugauccagagggugcagucgaauaagauacagagggaccauccacaguaccuacucucaucacaaucuuauucuucuccucacuuauuauuuuucugaucauuuucuuuuacucuauugaugaaguguuauguuuugauucuu
......................uggaucuuguuugacagcacuN............................................................................................................................................................................................................    4      1    LT-2
......................uggaucuugCuugacagcacuc............................................................................................................................................................................................................    1      1    JCK-3
......................uggaucuuguuugacagcacuU............................................................................................................................................................................................................    64     1    LCK-3
......................uggaucuuguuugacagcacuU............................................................................................................................................................................................................    68     1    JCK-1
......................uggaucuuguuugacCgcacuc............................................................................................................................................................................................................    5      1    JCK-1
......................uggaucuuguuugacagUacuc............................................................................................................................................................................................................    1      1    JCK-1
......................uggaucuuguuugacagcGcuc............................................................................................................................................................................................................    3      1    JCK-2
......................uggaucuuguuugCcagcacuc............................................................................................................................................................................................................    2      1    JT-1
......................uggaucuuguuugacagcacuA............................................................................................................................................................................................................    38     1    JCK-1
......................uggCucuuguuugacagcacuc............................................................................................................................................................................................................    4      1    LT-1
......................uggaucuuguuugacagcacNc............................................................................................................................................................................................................    3      1    JCK-1
......................uggGucuuguuugacagcacuc............................................................................................................................................................................................................    1      1    JCK-1
......................uggaucCuguuugacagcacuc............................................................................................................................................................................................................    1      1    JCK-1
......................uggaucuuguuuAacagcacuc............................................................................................................................................................................................................    1      1    JCK-1
......................uggaucuuguuugacagcacuU............................................................................................................................................................................................................    18     1    LCK-1
......................uggaucuuguuugacagcCcuc............................................................................................................................................................................................................    9      1    JCK-3
......................uggaucuCguuugacagcacuc............................................................................................................................................................................................................    3      1    JCK-2
......................uggaucuuguuuUacagcacuc............................................................................................................................................................................................................    1      1    JCK-1
......................uggaucuuguuGgacagcacuc............................................................................................................................................................................................................    3      1    JCK-1
......................uggaucuuguuugacagcacuG............................................................................................................................................................................................................    1      1    LT-2
......................uggaucuugCuugacagcacuc............................................................................................................................................................................................................    1      1    JCK-2
......................uggaucuuguuugacagcacuU............................................................................................................................................................................................................    1      1    JT-2
......................uggaucuuguuugacagcacGc............................................................................................................................................................................................................    1      1    JCK-1
......................uggaucuuAuuugacagcacuc............................................................................................................................................................................................................    4      1    JCK-3
......................ugAaucuuguuugacagcacuc............................................................................................................................................................................................................    4      1    JCK-2
......................uggauUuuguuugacagcacuc............................................................................................................................................................................................................    1      1    JCK-3
......................uggCucuuguuugacagcacuc............................................................................................................................................................................................................    7      1    LT-3
......................uggaucuuguuugacagcaUuc............................................................................................................................................................................................................    3      1    LT-2
......................uggaucuuguuugacagcacuc............................................................................................................................................................................................................    152    0    JT-3
......................uggaucuuguuugacagcacuU............................................................................................................................................................................................................    47     1    LT-1
......................uggaucCuguuugacagcacuc............................................................................................................................................................................................................    5      1    JCK-3
......................uggCucuuguuugacagcacuc............................................................................................................................................................................................................    2      1    LCK-1
......................uggaucuuguuugacagcaUuc............................................................................................................................................................................................................    8      1    JCK-3
......................uggaucuuguuugaAagcacuc............................................................................................................................................................................................................    4      1    JCK-2
......................uggaucuuguuugGcagcacuc............................................................................................................................................................................................................    3      1    JCK-2
......................uggaucuugGuugacagcacuc............................................................................................................................................................................................................    3      1    JCK-2
......................uggaucuuguuugCcagcacuc............................................................................................................................................................................................................    2      1    LT-3
......................Cggaucuuguuugacagcacuc............................................................................................................................................................................................................    6      1    JCK-2
......................uggaucuuguuugacagcacuc............................................................................................................................................................................................................    1069   0    LT-3
......................uggauUuuguuugacagcacuc............................................................................................................................................................................................................    5      1    JCK-1
......................ugCaucuuguuugacagcacuc............................................................................................................................................................................................................    1      1    JCK-2
......................ugNaucuuguuugacagcacuc............................................................................................................................................................................................................    2      1    LT-2
......................uggaucuuguuugacagcacuG............................................................................................................................................................................................................    2      1    JCK-3
......................uUgaucuuguuugacagcacuc............................................................................................................................................................................................................    1      1    LT-2
......................uggaucuuguuugaGagcacuc............................................................................................................................................................................................................    4      1    JCK-2
......................uggaucuuguCugacagcacuc............................................................................................................................................................................................................    1      1    JCK-1
......................ugAaucuuguuugacagcacuc............................................................................................................................................................................................................    1      1    LT-2
......................uNgaucuuguuugacagcacuc............................................................................................................................................................................................................    17     1    JCK-2
......................uggaucuuguuugacagcacGc............................................................................................................................................................................................................    1      1    JCK-3
......................uggaucuuguuugacCgcacuc............................................................................................................................................................................................................    13     1    JCK-2
......................uggaucuuguuugacagcacuA............................................................................................................................................................................................................    41     1    JCK-3
......................uAgaucuuguuugacagcacuc............................................................................................................................................................................................................    1      1    LCK-3
......................uggaucuuguuugacCgcacuc............................................................................................................................................................................................................    1      1    LT-2
......................uggaucCuguuugacagcacuc............................................................................................................................................................................................................    2      1    JCK-2
......................uggaucuuguuugacagNacuc............................................................................................................................................................................................................    1      1    JCK-3
......................uggaucuuguuugacagcaUuc............................................................................................................................................................................................................    5      1    JCK-2
......................uggaucuuguuugacagcacuA............................................................................................................................................................................................................    11     1    JT-1
......................uggaucuugGuugacagcacuc............................................................................................................................................................................................................    3      1    JCK-1
......................uggaucuuguuugacagcacuc............................................................................................................................................................................................................    12872  0    JCK-3
......................uggaucuuguuugacagcacuUu...........................................................................................................................................................................................................    71     1    LT-2
......................uggaucuuguuugacagcacucG...........................................................................................................................................................................................................    5      1    JCK-2
......................uggaucuuguuugacagcacucA...........................................................................................................................................................................................................    79     1    JCK-1
......................uggaucuuguuugacagcacuUu...........................................................................................................................................................................................................    112    1    JCK-3
......................uggaucuuguuugacagcacucA...........................................................................................................................................................................................................    100    1    JCK-3
......................uggaucuuguuugacagcacuAu...........................................................................................................................................................................................................    1      1    JCK-3
......................uggaucuuguuugacagcacuUu...........................................................................................................................................................................................................    23     1    LCK-1
......................uggaucuuguuugacagcacucu...........................................................................................................................................................................................................    26     0    LT-1
......................uggaucuuguuugacagcacuUu...........................................................................................................................................................................................................    19     1    LCK-2
......................uggaucuuguuugacagcacuUu...........................................................................................................................................................................................................    21     1    JT-3
......................uggaucuuguuugacagcacuGu...........................................................................................................................................................................................................    1      1    LT-3
```

Mature Star

```
uccaucuuuuucguuuacucucuggaucuuguuugacagcacucuccuauauuuuaccauucaucuuuaaaugcgaaauggcgaugaugaugggagugcugucaacaugauccagagggugcagucgaauaagauacagagggaccauccacaguaccuacucucaucacaaucuuauucuucuccucacuuauuauuuuucugaucauuuucuuuuacucuauugaugaaguguuauguuuugauucuu
......................uggaucuuguuugacagcacuAu      3    1    JCK-1
......................uggaucuuguuugacagcacuUu      21   1    JT-2
......................uggaucuuguuNgacagcacucu      3    1    JCK-1
......................uggaucuuguuugacagcacuAu      2    1    JCK-2
......................uggaucuuguuugacagcacuUu      62   1    LT-1
......................uggaucuuguuugacagcacucG      4    1    JCK-3
......................uggaucuuguuugacagcacucu      16   0    LCK-3
......................uggaucuuguuugacagcacucG      3    1    JCK-1
......................uggaucuuguuugacagcacucu      90   0    JCK-1
......................uggaucuuguuugacagcacuGu      2    1    LT-2
......................uggaucuuguuugacagcacuGu      5    1    LT-1
......................uggaucuuguuugacagcacucu      111  0    JCK-2
......................uggaucuuguuugacagcacuUu      75   1    LCK-3
......................uggaucuuguuugacagcacucA      24   1    LCK-3
......................uggaucuuguuugacagcacuUu      53   1    LT-3
......................uggaucuuguuugacagcacucA      140  1    JCK-2
......................uggaucuuguuugacagcacucA      14   1    LT-2
......................uggaucuuguuugacagcacuGu      6    1    JCK-2
......................uggaucuuguuugacagcacuUu      39   1    JT-1
......................uggaucuuguuugacagcacucA      23   1    LT-1
......................uggaucuuguuugacagcacucA      10   1    JT-2
......................uggaucuuguuugacagcacuGu      1    1    JCK-1
......................uggaucuuguuugacagcacucA      14   1    JT-3
......................uggaucuuguuugacagcacuUu      109  1    JCK-1
......................uggaucuuguuugacagcacucu      80   0    JCK-3
......................uggaucuuguuugacagcacuUu      159  1    JCK-2
......................uggaucuuguuugacagcacucu      14   0    LT-2
......................uggCucuuguuugacagcacucu      1    1    LT-2
......................uggaucuuguuugacagcacucA      6    1    LT-3
......................uggaucuuguuugacagcacucu      10   0    LT-3
......................uggaucuuguuugacagcacuUuc     3    1    JCK-2
......................uggaucuuguuugacagcacucuA     1    1    LT-1
......................uggaucuuguuugacagcacucAc     2    1    LT-3
......................uggaucuuguuugacagcacucuc     1    0    LT-1
......................uggaucuuguuugacagcacucuA     2    1    JCK-2
......................uggaucuuguuugacagcacucuA     2    1    JCK-3
......................uggaucuuguuugacagcacuGuc     1    1    LT-3
......................uggaucuuguuugacagcacucuA     5    1    LT-2
......................uggaucuuguuugacagcacucuU     7    1    LT-2
......................uggaucuuguuugacagcacucuU     6    1    JCK-2
......................uggaucuuguuugacagcacucuc     19   0    JCK-2
......................uggaucuuguuugacagcacucuc     1    0    LT-3
......................uggaucuuguuugacagcacucuc     19   0    JCK-3
......................uggaucuuguuNgacagcacucuc     1    1    JCK-1
......................uggaucuuguuugacagcacucuA     1    1    LT-3
......................uggaucuuguuugacagcacucuU     1    1    JCK-1
......................uggaucuuguuugacagcacucuc     12   0    JCK-1
......................uggaucuuguuugacagcacucAc     7    1    LT-1
......................uggaucuuguuugacagcacucuc     3    0    LCK-3
......................uggaucuuguuugacagcacucuA     2    1    JCK-1
......................uggaucuuguuugacagcacucAc     13   1    LCK-3
......................uggaucuuguuugacagcacucuU     9    1    LT-1
......................uggaucuuguuugacagcacucuccu   2    0    JCK-2
......................uggaucuuguuugacagcacucuccu   2    0    JCK-1
......................uggaucuuguuugacagcacucuccu   2    0    JCK-3
.......................ggaucuuguuugacagca          2    0    JCK-3
.......................ggaucuuguuugacagca          1    0    JCK-1
.......................ggaucuuguuugacagca          1    0    LT-1
.......................ggaucuuguuugacagcac         4    0    LCK-3
.......................ggaucuuguuugacagcac         2    0    LT-1
.......................ggaucuuguuugacagcac         1    0    LT-2
.......................ggaucuuguuugacagcacu        5    0    LT-2
.......................ggaucuuguuugacagcacu        4    0    JCK-3
.......................ggaucuuguuugacagcacu        2    0    LT-3
.......................ggaucuuguuugacagcacu        2    0    LCK-3
.......................ggaucuuguuugacagcacu        5    0    JCK-1
.......................ggaucuuguuugacagcacu        3    0    JCK-2
.......................ggaucuuguuugacagcacu        5    0    LT-1
.......................ggaucuuguuugacagcacuc       6    0    JCK-2
.......................ggaucuuguuugacagcacuc       14   0    JCK-3
```

Mature · **Star**

uccaucuuuuucguuuacucucuggaucuuguuugacagcacucuccuauauuuuaccauucaucuuuaaaugcgaaauggcgaugaugaugggagugcugucaacaugauccagagggugcagucgaauaagauacagagggaccauccacaguaccuacucucaucacaaucuuauucuucuccucacuuauuauuuuucugaucauuuucuuuuacucuauugaugaaguguuauguuuugauucuu

ggaucuuguuugacagcacuc	6	0	JCK-1
ggaucuuguuugacagcacucu	5	0	JCK-1
ggaucuuguuugacagcacucu	4	0	JCK-3
ggaucuuguuugacagcacucA	2	1	JCK-2
ggaucuuguuugacagcacucu	6	0	JCK-2
gaucuuguuugacagcacu	3	0	LT-3
gaucuuguuugacagcacu	14	0	LT-2
gaucuuguuugacagcacu	1	0	JCK-2
gaucuuguuugacagcacu	1	0	LT-1
gaucuuguuugacagcacu	3	0	JCK-1
gaucuuguuugacagcacuc	2	0	LT-1
gaucuuguuugacagcacuc	3	0	LT-3
gaucuuguuugacagcacucu	1	0	LT-1
gaucuuguuugacagcacucA	1	1	LT-1
gaucuuguuugacagcacucuc	1	0	JCK-1
gaucuuguuugacagcacucuc	4	0	JCK-2
gaucuuguuugacagcacucuc	1	0	JCK-3
aucuuguuugacagcacu	2	0	LT-1
aucuuguuugacagcacu	3	0	LT-2
aucuuguuugacagcacuU	1	1	LT-2
aucuuguuugacagcacuc	1	0	LT-2
aucuuguuugacagcacuc	2	0	LT-1
aucuuguuugacagcacuc	5	0	LT-3
aucuuguuugacagcacuc	1	0	JCK-2
aucuuguuugacagcacuc	8	0	LCK-3
aucuuguuugacagcacucu	24	0	LCK-3
aucuuguuugacagcacucu	15	0	JCK-2
aucuuguuugacagcacucu	3	0	JCK-1
aucuuguuugacagcacucu	4	0	JCK-3
aucuuguuugacagcacucu	4	0	LT-1
aucuuguuugacagcacucu	2	0	LT-2
aucuuguuugacagcacucA	1	1	LT-2
aucuuguuugacagcacucA	2	1	LT-1
aucuuguuugacagcacucA	7	1	LCK-3
aucuuguuugacagcacuUu	1	1	LT-1
aucuuguuugCcagcacucu	1	1	JCK-2
aucuuguuugacagcacucu	4	0	LT-3
aucuuguuugacagcacuUu	1	1	LT-3
aucuuguuugacagcacucuc	11	0	JCK-3
aucuuguuugacagcacucuA	1	1	JCK-3
aucuuguuugacagcacucuU	4	1	LT-2
aucuuguuugacagcacucuA	3	1	JCK-1
aucuuguuugacagcacucuc	8	0	JCK-1
aucuuguuugacagcacucuU	6	1	LCK-3
Cucuuguuugacagcacucuc	1	1	JCK-2
aucuuguuugacagcacucuc	12	0	JCK-2
aucuuguuugacagcacucucc	2	0	JCK-1
aucuuguuugacagcacucuUc	3	1	LT-2
aucuuguuugacagcacucucG	1	1	JCK-1
aucuuguuugacagcacucucc	1	0	JCK-3
ucuuguuugacagcacuc	2	0	LT-3
ucuuguuugacagcacuc	1	0	LT-2
ucuuguuugacagcacucu	2	0	JT-2
ucuuguuugacagcacucA	1	1	LT-3
ucuuguuugacagcacucu	2	0	JCK-2
ucuuguuugacagcacucu	2	0	LT-1
ucuuguuugacagcacucuc	2	0	JCK-2
ucuuguuugacagcacucuc	1	0	JCK-3
gaugaugaugggagugcugu	1	0	JCK-2
gaugaugaugggagugcugu	1	0	JCK-1
gaAgggagugcugucaacaugauc	1	1	JCK-3
agugcugucaacaugauccaga	2	0	JCK-1
agugcugucaacaugauccaga	4	0	JCK-3
agugcugucaacaugauccaga	3	0	JCK-2
gugcugucaacaugaucc	1	0	JCK-2
gugcugucaacaugauccaga	32	0	JCK-2
gugcugucaacaugauccaga	1	0	LT-1
gugcugucNacaugauccaga	1	1	JCK-3
gugcugucaacaugauccaga	9	0	JCK-1
gugcugucaacaugauccaga	20	0	JCK-3

```
                      Mature                                                                         Star
uccaucuuuuucguuuacucucuggaucuuguuugacagcacucuccuauauuuuaccauucaucuuuaaaugcgaaauggcgaugaugaugggagugcugucaacaugauccagagggugcagucgaauaagauacagagggaccauccacaguaccuacucucaucacaaucuuauucuucuccucacuuauuauuuuucugaucauuuucuuuuacucuauugaugaaguguuauguuuugauucuu
...............................................................................................gugcugucaacaugauccagaA.....................................................................................................................................   5   1   JCK-2
...............................................................................................gugcugucGacaugauccagag.....................................................................................................................................   1   1   LT-1
```
